# Characterization of ADAMTS9 proteoglycanase activity: Comparison with ADAMTS1, ADAMTS4, and ADAMTS5

**DOI:** 10.1016/j.jbc.2025.110301

**Published:** 2025-05-29

**Authors:** Daniel R. Martin, Gemma Sardelli, Tina Burkhard, Milan M. Fowkes, Alexander F. Minns, Roberta Moschini, Antonella Del Corso, Rens de Groot, Suneel S. Apte, Salvatore Santamaria

**Affiliations:** 1 Department of Biomedical Engineering, Cleveland Clinic Research, Cleveland, Ohio, USA; 2 Department of Biology, Biochemistry Unit, University of Pisa, Pisa, Italy; 3 Department of Biochemical Sciences, School of Biosciences, Faculty of Health and Medical Sciences, University of Surrey, Surrey, United Kingdom; 4 Centre for Medicines Discovery, University of Oxford, Oxford, United Kingdom; 5 Institute of Cardiovascular Science, University College London, London, United Kingdom; 6 Department of Immunology and Inflammation, Imperial College London, London, United Kingdom

**Keywords:** proteoglycans, aggrecan, versican, biglycan, ADAMTS, metalloprotease

## Abstract

A Disintegrin-like And Metalloprotease domain with Thrombospondin type I motifs (ADAMTS) 9 has essential, non-redundant roles during embryogenesis. *Adamts9* null murine embryos die prior to completing gastrulation. Unusually, for a protease, *Adamts9* haploinsufficiency results in cardiovascular and ocular anomalies. ADAMTS9 is required for proteostasis of versican, a widely distributed large aggregating proteoglycan abundant in the provisional extracellular matrix during embryogenesis. Despite its importance, ADAMTS9 proteoglycanase activity has undergone limited characterization, especially in comparison to ADAMTS1, ADAMTS4, and ADAMTS5, due to difficulties in expressing and purifying the >200 kDa full-length form of ADAMTS9. Like ADAMTS1, ADAMTS4, and ADAMTS5, ADAMTS9 cleaves versican V1 isoform at E441-A442, but unlike them, cleavages at other sites are unknown. Here, we expressed a truncated ADAMTS9 construct (ADAMTS9 MDTCS) consisting of all ADAMTS “core domains” present in ADAMTS1, ADAMTS4, and ADAMTS5, and characterized its activity against versican, aggrecan, and the small leucine-rich proteoglycan biglycan. We identified cleavages in versican (V1 and V2 isoforms) and biglycan using a *z*-score approach based on label-free quantitation of semi- and fully tryptic/GluC peptides. Moreover, using a quantitative assay, we established that ADAMTS9 MDTCS versicanase activity at the E441-A442 site is 165-fold lower than ADAMTS5, 9-fold lower than ADAMTS4, and 6-fold higher than ADAMTS1. Finally, we confirmed that ADAMTS9 MDTCS cleaves bovine aggrecan at E392-A393. This analysis of the proteoglycanase activity in the ADAMTS family highlights differences and similarities in cleavage site specificities which could be leveraged to develop selective small molecule inhibitors against current targets of interest, ADAMTS4, ADAMTS5, and ADAMTS7.

The mammalian ADAMTS family comprises 19 genes encoding secreted multidomain metalloproteases (1). ADAMTS proteases have a conserved domain organization consisting of the N-terminal prodomain, a zinc metalloprotease (Mp) domain, a disintegrin-like domain (Dis), a thrombospondin type 1 motif/repeat (TSR), a cysteine-rich (CR) domain, and a spacer (Sp) domain. These domains alone comprise the complete structure of ADAMTS4, whereas all other ADAMTSs have additional C-terminal ancillary (non-catalytic) domains. For example, ADAMTS9, the largest mammalian ADAMTS, has 14 additional TSRs and a unique C-terminal GON-1 domain (Fig. 1*A*) (2). ADAMTS9 is also one of two most highly conserved ADAMTSs (the other being its homolog ADAMTS20), with readily recognizable orthologs in the nematode *Caenorhabditis elegans* (*gon-1*) (2), the fruit fly *Drosophila melanogaster* (Adamts-A) (3), and the basal chordate *Ciona intestinalis* (Ciona677) (4). In *C. elegans*, mutations in *gon-1* cause severe gonadal anomalies due to failed germ cell migration, resulting in recessive, fully penetrant sterility (5, 6). In *Danio rerio* (zebrafish), loss of Adamts9 is associated with reduced fertility due to impaired ovarian development (7) and spine defects (8). While the inactivation of ADAMTS9 orthologs is compatible with life in nematodes (5, 6) and zebrafish (7, 9), *Adamts9* null mouse embryos die before completing gastrulation (10, 11, 12). Mice with *Adamts9* haploinsufficiency reach adulthood but display cardiac and aortic anomalies (13), spontaneous corneal neovascularization (14) and congenital corneal opacity of developmental origin (15) with variable penetrance. A hypomorphic *Adamts9* mutation and conditional gene inactivation have revealed important roles for ADAMTS9 in regulating umbilical cord growth, uterine contraction during parturition, limb development, and neural tube closure (16, 17, 18, 19). These phenotypes indicate that proper dosage of ADAMTS9 is required during early-to mid-embryogenesis, consistent with *Adamts9* expression during the formation of the three germ layers and in many of their derivative cell populations (12, 20). In adult murine tissues, ADAMTS9 is expressed by microvascular endothelial cells (14, 17), skin fibroblasts and melanoblasts (2, 21), myometrial and umbilical cord smooth muscle cells (17, 18), skeletal muscle (2, 14), and articular chondrocytes (22). Genome-wide association studies have implicated the *ADAMTS9* locus in type II diabetes (23), obesity (24), and macular degeneration (25). Recessive missense mutations in the *ADAMTS9* locus cause two diseases characterized by impaired formation of the primary cilium, nephronophthisis-related ciliopathy (9), and Joubert syndrome (26). Phenotypes resembling human ciliopathies are also evident in *Adamts9/Adamts20* double mutant mice (which survive to 15 days of gestation if they include the hypomorphic, but not the inactivated *Adamts9* allele) (19). ADAMTS9 and ADAMTS20 act cooperatively in key developmental processes such as interdigital web regression (10), skin pigmentation (21, 27), and palate closure (11).

**Figure 1:**
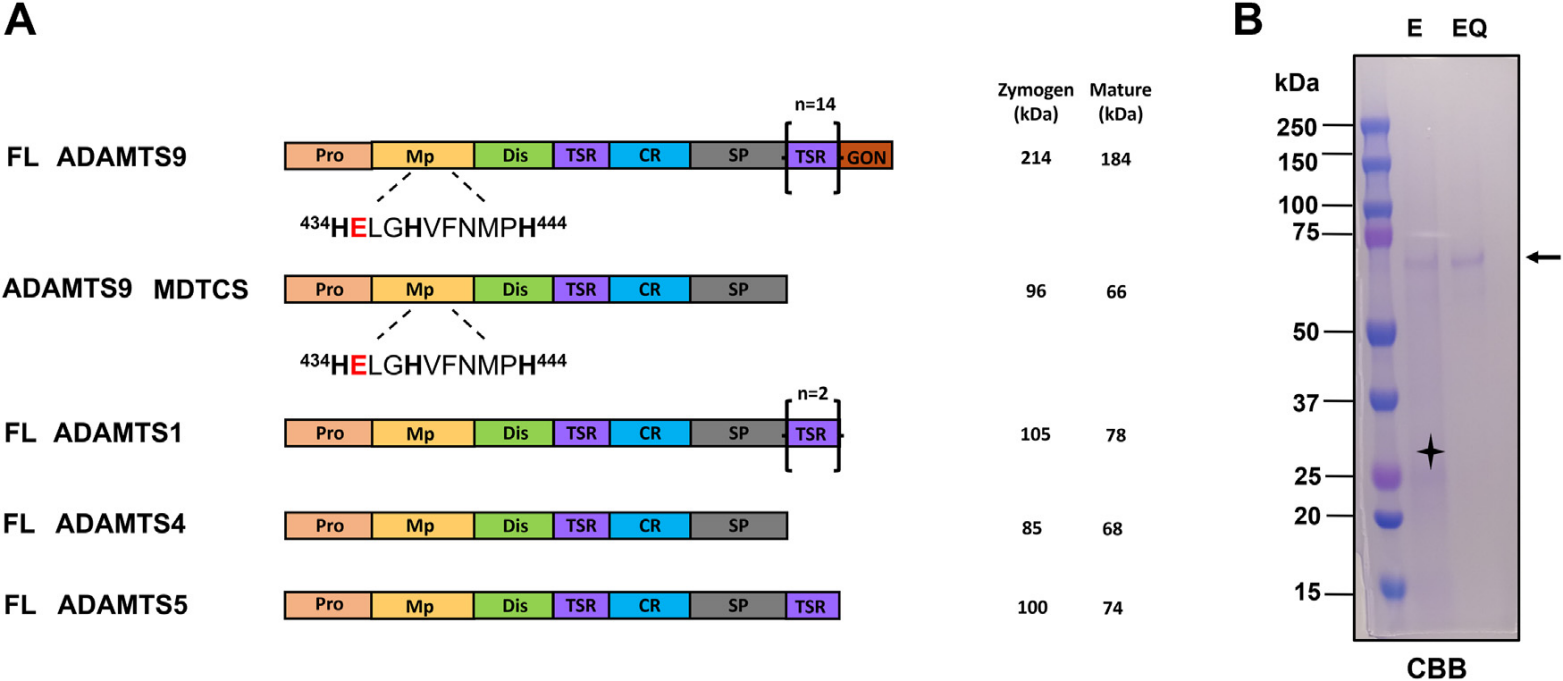
**Expression and purification of ADAMTS9 MDTCS.***A*, schematic of secreted full-length (FL) ADAMTS9 and ADAMTS9 MDTCS alongside full-length ADAMTS1, ADAMTS4, and ADAMTS5. The zinc binding sequence (residues 434–444) in ADAMTS9 is shown below the *cartoon*. Histidine residues involved in zinc coordination are in *bold*, the active site glutamic acid residue (E435) mutated in ADAMTS9 EQ is shown in *red*. Molecular weights of zymogen (*i.e.*, prodomain-containing) and mature (*i.e.*, prodomain-lacking) forms were calculated using Expasy ProtParam (https://web.expasy.org/protparam/). The signal peptide at the N-terminus and the FLAG tag at the C-terminus are not shown. Domains are not drawn to scale. *B*, Coomassie Brilliant Blue (CBB) staining of active (E) and inactive (EQ) ADAMTS MDTCS. The *arrow* indicates the mature form at ∼70 kDa, the *asterisk* the autolytic product at ∼25 kDa. CR, cysteine-rich domain; Dis, disintegrin-like domain; Mp, metalloprotease domain; Pro, prodomain; Sp, spacer domain; TSR, thrombospondin type I motif.

ADAMTS9 clusters phylogenetically with ADAMTS20 in a distinct clade, suggesting an origin before divergence of deuterostomes and protostomes, in relative evolutionary proximity to ADAMTS1, ADAMTS4, ADAMTS5, ADAMTS8, and ADAMTS15 (1). Despite evolutionary distance, the two clades share the ability to cleave the large aggregating proteoglycans/hyalectans aggrecan and versican at canonical sites (2). Hyalectans are characterized by the presence of an N-terminal globular domain (G1) that binds to the unsulfated glycosaminoglycan (GAG) hyaluronan, an extended central region highly decorated with chondroitin/dermatan sulfate GAGs as well as *N*- and *O*-linked sugars, and a C-terminal globular domain (G3) that binds other ECM molecules.

Versican is an essential component of newly synthesized and transient (‘provisional’) extracellular matrix (ECM), where it contributes to tissue swelling and regulates cell differentiation, proliferation, adhesion, and migration (28). In addition to its N- and C-terminal globular domains (named G1 and G3, respectively), versican contains a central, GAG-rich region consisting of one or both GAGα and GAGβ domains, which are encoded by distinct exons. Alternative splicing of these exons generates four canonical versican isoforms: V0 (containing both GAG domains), V1 (containing only GAGβ), V2 (containing only GAGα), and V3 (lacking both GAG domains) (28). While V0, V1, and V3 mRNAs are detected in most tissues, V2 is mostly expressed in the central nervous system (29). ADAMTS9 has biochemically demonstrated versicanase activity that is corroborated by the phenotypes of *Adamts9*-deficient mice, which show versican accumulation associated with reduced versican cleavage (10, 13, 17, 18, 19).

So far, the proteoglycanase activity of ADAMTS9 has not been further characterized nor directly compared to that of other ADAMTSs. Full-length ADAMTS9 (Fig. 1*A*) is characterized by high molecular weight (∼210 kDa), low expression levels, susceptibility to autoproteolysis, and association with the cell surface and ECM, which have so far prevented its purification (2, 14, 30, 31, 32). To circumvent these challenges, we expressed a truncated recombinant ADAMTS9 comprising the core ADAMTS domains and characterized its proteoglycanase activity.

## Results

### Expression and purification of a truncated ADAMTS9 construct

We designed a construct truncated after the Sp domain (ADAMTS9 MDTCS) (Fig. 1*A*), as this is the domain composition of the smallest ADAMTS, ADAMTS4, and other ADAMTS proteases, which were similarly truncated, efficiently expressed, and purified (33, 34, 35, 36). Furthermore, ADAMTS9 MDTCS retains all domains that were previously determined in ADAMTS1, ADAMTS4, and ADAMTS5 to be necessary for proteoglycanase activity (35, 37), thus allowing a comparison of ADAMTS9 with the most closely related ADAMTS proteases. Using ADAMTS9 MDTCS as a template, a control construct coding for catalytically inactive ADAMTS9 (ADAMT9 EQ) was generated by mutating the active site glutamic acid (E435) to glutamine (Fig. 1*A*). Both constructs, expressed in HEK293T cells in the presence of heparin to release ECM- and cell surface-bound ADAMTS9 (2, 19), were purified as demonstrated by SDS-PAGE and Coomassie brilliant blue (CBB) staining (Fig. 1*B*). Both proteins migrated as major bands of ∼70 kDa, consistent with the predicted size of the mature protease resulting from furin excision of the prodomain (Fig. 1*A*). An additional band of ∼25 kDa and a faint smear were observed only in the active (E) construct (Fig. 1*B*), suggesting an autolytic origin.

### Identification of autolytic cleavage sites in ADAMTS9 MDTCS

**Figure 2:**
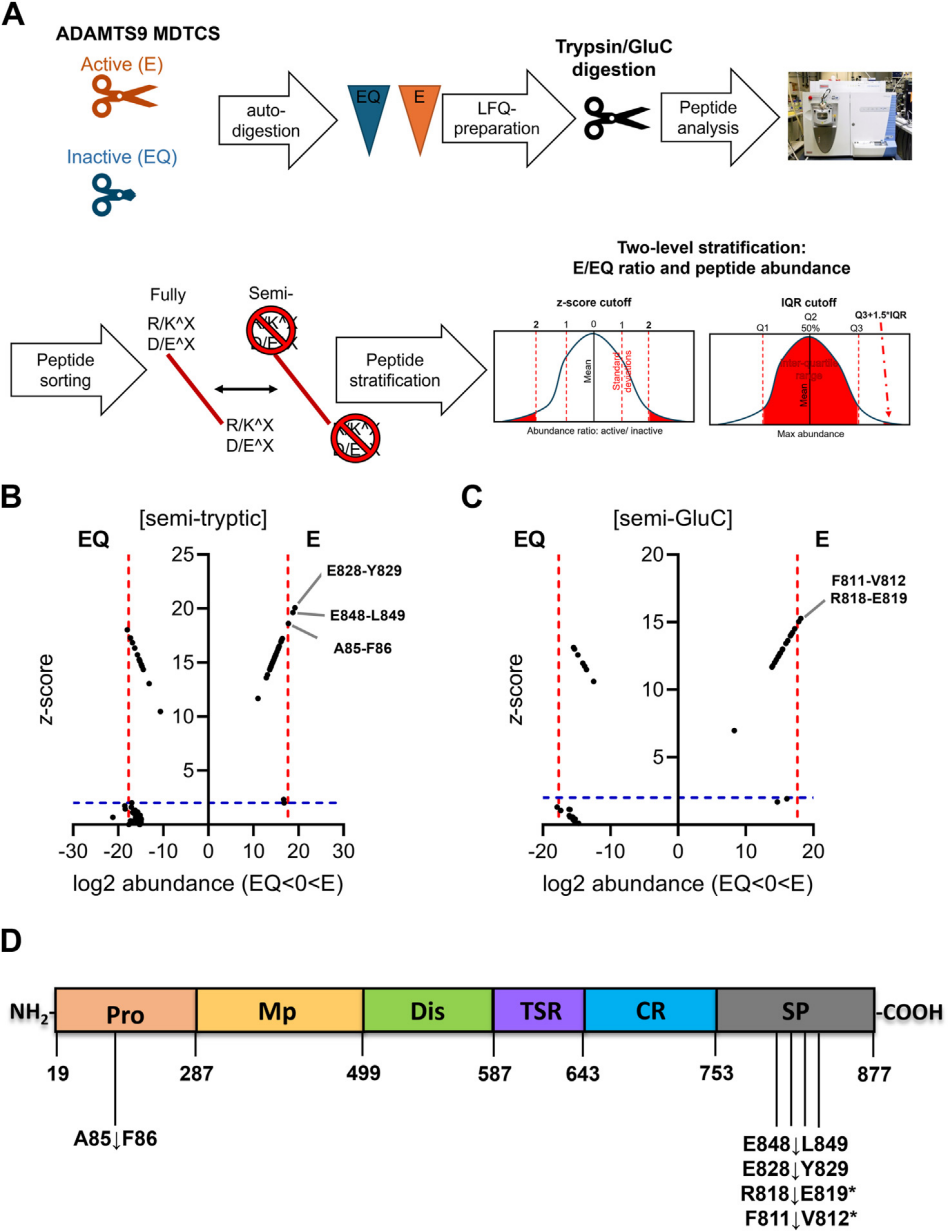
**Identification of autolytic cleavage sites in ADAMTS9 MDTCS using label-free quantitative proteomics and *z*-score analysis.***A*, schematic of the general experimental design. Active (E) and inactive (EQ) ADAMTS9 MDTCS were incubated separately and digested with trypsin or GluC prior to LC-MS/MS. Significant peptides were identified using a two-step analysis. LFQ, label-free quantitative proteomics. *B* and *C*, plots of log2 peptide abundance *versus z*-score for semi-tryptic (*B*) and semi-GluC (*C*) peptides. *Dashed blue* and *red lines* indicate the thresholds for *z*-score and log2 peptide abundance, respectively. Autolytic sites are indicated. *D*, overview of autolytic sites in ADAMTS9. *Asterisks* indicate cleavage sites identified in digests treated with GluC. Numbering according to UniProt ID Q9P2N4-3. Domains are not drawn to scale. CR, cysteine-rich domain; Dis, disintegrin-like domain; Mp, metalloprotease domain; Pro, prodomain; Sp, spacer domain; TSR, thrombospondin-like motif.

As CBB staining of ADAMTS9 MDTCS showed low-molecular-weight fragments only in the catalytically competent E construct (Fig. 1*B*), we applied label-free quantitative (LFQ) proteomics followed by *z*-score analysis as previously described (38) to identify putative autolytic sites in ADAMTS9 MDTCS. ADAMTS9 MDTCS E and EQ were separately incubated at 37 °C for 2 h, then subjected to trypsin digestion, and analyzed by liquid chromatography-tandem mass spectrometry (LC-MS/MS). GluC, which cleaves at the C-terminus of glutamic acid and aspartic acid residues, was used as an alternative (Fig. 2*A*) due to the presence of 38 GluC-susceptible bonds in ADAMTS9 MDTCS ancillary domains (excluding the FLAG tag). *Z*-scores were calculated to define outlier semi-tryptic/GluC and fully tryptic/GluC E/EQ ratios indicative of ADAMTS9-mediated proteolysis. Specifically, in addition to semi-tryptic/GluC peptides with significant *z*-scores, we analyzed the relative abundance of fully tryptic/fully GluC peptides spanning the putative cleavage sites. Significant cleavage events were subsequently stratified by peptide abundance using a cut-off of 1 + 1.5 interquartile range. Fully tryptic/GluC peptides having a significant *z*-score in the EQ strongly supported cleavages occurring within them, as previously described (38).

One-hundred-seventy-seven positionally internal semi-tryptic and 55 semi-GluC peptides were identified, covering 76% of the ADAMTS9 MDTCS sequence. Of these, five were significantly more abundant in the E than in the EQ auto-digests (Fig. 2, *B* and *C*), suggesting five autolytic sites, of which 4 occurred in the Sp domain and one in the prodomain (Table 1 and Fig. 2*D*). However, none of these cleavage sites were predicted to generate a fragment matching in size the 25 kDa band observed specifically in our ADAMTS9 E preparations (Fig. 1*B*). No fully tryptic or fully GluC peptides were significantly more abundant in the EQ than in the E auto-digests (Fig. S1).

**Table 1:** ADAMTS9 peptides significantly more abundant in ADAMTS9 MDCTS auto-digests

Semi-tryptic or semi-GluC peptide	Working protease	Peptide parameters			
		Cleavage site position	Domain	Log2 abundance	*Z*-score
**[A].F**ASSSSSSTSSQAHYR.[L]	Tryp	A85-F86	Pro	17.8	18.6
**[F].V**VTMAKRE.[I]	GluC	F811-V812	Sp	17.9	15.0
**[R].E**IRIGNAVVE.[Y]	GluC	R818-E819	Sp	18.1	15.3
**[E].Y**SGSETAVER.[I]	Tryp	E828-Y829	Sp	19.2	20.1
**[E].L**LLQVLSVGK.[L]	Tryp	E848-L849	Sp	18.8	19.6

Residues within brackets precede or follow the detected peptide sequence (within periods). P1 and P1′ residues are in bold. *Z*-score is the number of standard deviations from the mean E/EQ ratio of all peptides found in both groups. Pro, prodomain; Sp, spacer domain; Tryp, trypsin. Residues are numbered according to UniProt ID Q9P2N4-3.

### Establishment of a quenched-fluorescent peptide cleavage assay for the determination of active ADAMTS9 concentration

**Figure 3:**
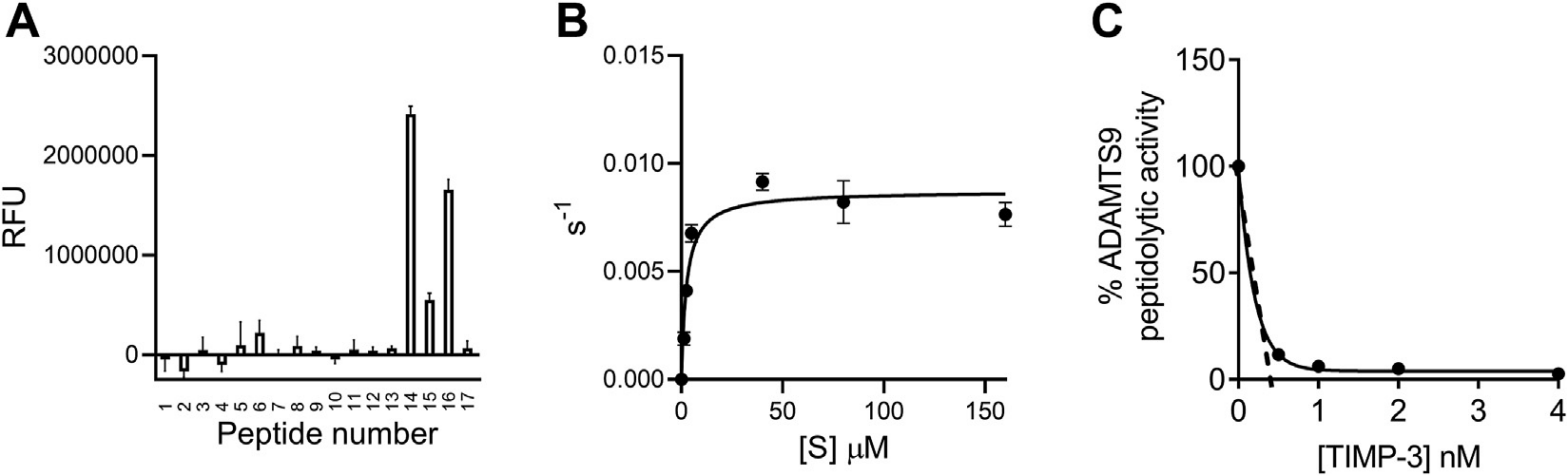
**Quenched fluorescent peptide cleavage assay for determination of active ADAMTS9 MDTCS concentration.***A*, screening of QF peptides as ADAMTS9 substrates. ADAMTS9 peptidolytic activity is reported as blank-subtracted Relative Fluorescence Units (RFU). Values are mean ± SD (n = 3 independent experiments). *B*, cleavage of peptide 14 by ADAMTS9 MDTCS (0.76 nM). Following correction for the inner filter effect, data were fitted to the Michaelis–Menten equation and are presented as average ± SD (n = 3 independent experiments). *C*, titration of ADAMTS9 MDTCS with TIMP-3. TIMP-3 (0–16 nM) was incubated with ADAMTS9 MDTCS (10 nM nominal concentration as measured by optical absorbance at 280 nm) at 37 °C for 1 h, before addition of QF peptide 14 (40 μM). Data on the y axis represent residual activity compared to uninhibited controls. A representative titration curve is shown, each point representing a mean of two technical replicates.

ADAMTS concentrations are generally determined by active site titration with Tissue Inhibitors of Metalloproteases (TIMPs), their endogenous inhibitors, in assays using quenched-fluorescent (QF) peptides as substrates (35, 37, 39, 40). Binding of TIMPs to ADAMTSs occurs in a 1:1 stoichiometric ratio through bidentate coordination of the protease catalytic zinc ion by the TIMP N-terminal cysteine residue (41). Compared to native substrates such as proteoglycans, QF peptides have the advantage that, being less than 15 amino acids in length, they do not engage distant binding sites (exosites) localized in the ADAMTS ancillary domains (42), allowing accurate active site quantification. To compare ADAMTS9 MDTCS proteoglycanase activity with that of ADAMTS1, ADAMTS4, and ADAMTS5, such an activity assay would be essential, but no QF peptide substrates have been reported for ADAMTS9. We therefore screened 17 QF peptides designed for ADAMTS5 (43), ADAMTS4 (44), and ADAMTS7 (39) as well as a general matrix metalloprotease substrate (45, 46) (Fig. 3*A* and Table S1). Peptide 14, a previously reported ADAMTS4 substrate (44), gave the highest background-corrected signal among the peptides tested and was selected for further analysis. At increasing concentrations of QF peptide 14, a Michaelis-Menten (*K*_m_) constant value of 2.6 ± 0.3 μM was obtained for ADAMTS9 MDTCS (Fig. 3*B*), approximately 10-fold lower than the *K*_m_ constant we previously reported for ADAMTS4 (46). As TIMP-3 is a tight binding inhibitor of ADAMTS1 (35), ADAMTS4, and ADAMTS5 (41), and was previously suggested as an ADAMTS9 inhibitor (22), concentrations of ADAMTS9 were determined by active-site titration with TIMP-3 (Fig. 3*C*). This allowed determination of both turnover number (*k*_cat_: 0.0087 ± 0.00068 s^−1^) and catalytic efficiency (*k*_cat_/*K*_m_: 3.4 ± 0.14 × 10^3^ M^−1^ s^−1^) (Fig. 3*B*), which were 126- and 1.6- fold lower, respectively, than those reported for ADAMTS4 (46).

### Characterization of ADAMTS9 MDTCS versicanase activity at the E441-A442 site in versican V1

**Figure 4:**
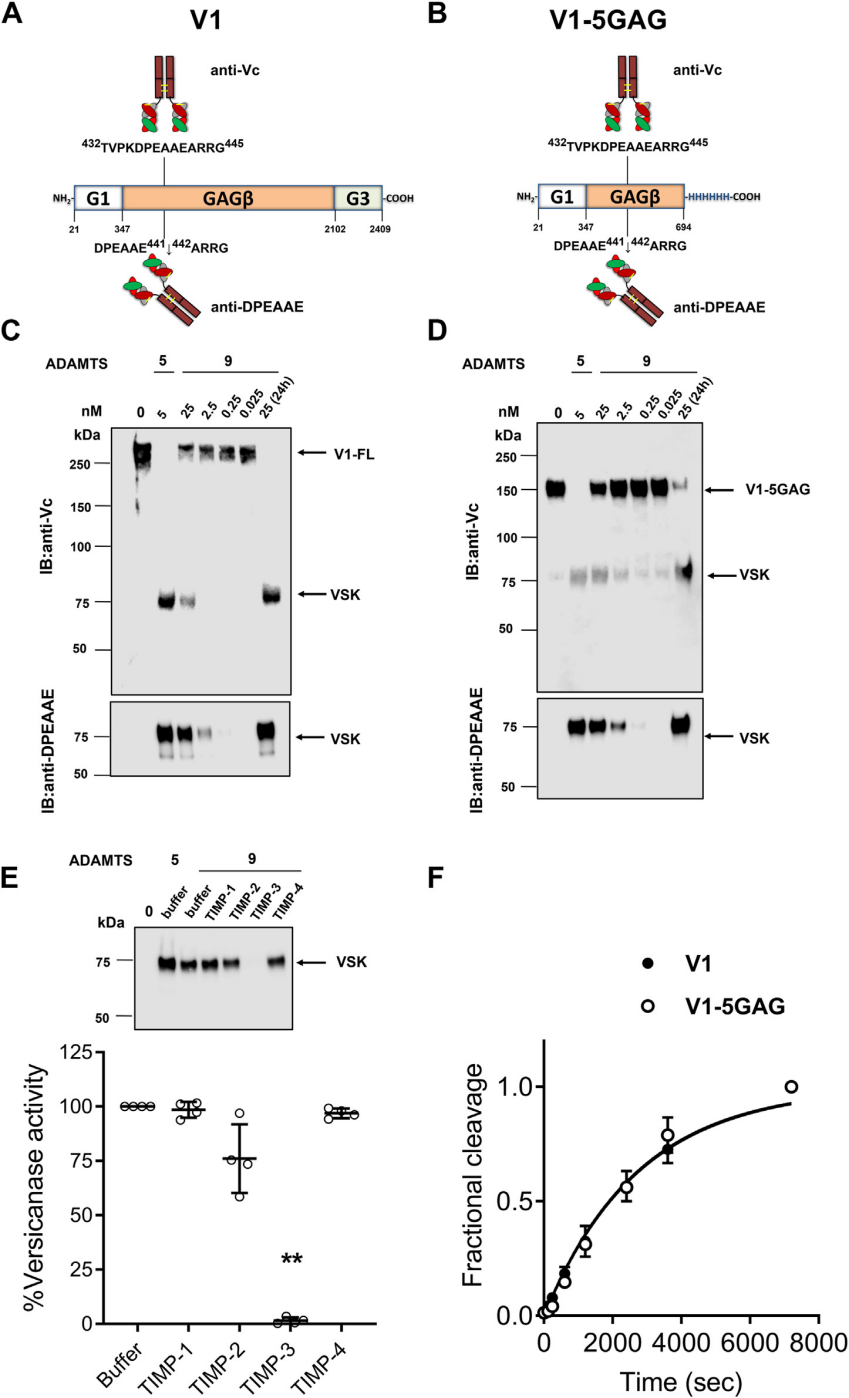
**Versicanase activity of ADAMTS9 MDTCS.***A* and *B*, schematic of domain structure and epitopes recognized by anti-DPEAAE and anti-Vc antibodies on V1 and V1-5GAG. *C* and *D*, ADAMTS9 MDTCS (0.025–25 nM) or full-length ADAMTS5 (5 nM) were incubated with 100 nM full length versican V1 (*C*) or V1-5GAG (*D*) for 2 h at 37 °C. Samples were deglycosylated, subjected to SDS-PAGE and immunoblotted either with anti-Vc or anti-DPEAAE. *E*, inhibitory activity of TIMPs against ADAMTS9 MDTCS. ADAMTS9 MDTCS (25 nM) was preincubated with TIMP-1, TIMP-2, TIMP-3, and TIMP-4 (each at 500 nM) or ADAMTS5 (5 nM) for 1 h at 37 °C before addition of 100 nM versican V1-5GAG. After 2 h, samples were deglycosylated, subjected to SDS-PAGE and immunoblotted for versikine (VSK) using anti-DPEAAE. Band intensities were quantified using ImageJ and statistically analyzed by GraphPad Prism Software. Results were reported as the mean of residual activities compared to ADAMTS9 versicanase activity in the absence of inhibitors (indicated as ‘buffer’) ± SD (n = 4 independent experiments). Statistical analysis was performed using one-way Anova with Dunn’s multiple comparisons. *p*∗∗ = 0.0031. For TIMP-1, TIMP-2 and TIMP-4 *p* values were >0.9999, 0.1058 and 0.9209, respectively. *F*, time course of ADAMTS9 MDTCS versicanase activity. ADAMTS9 MDTCS (25 nM) was incubated either with 50 nM V1 or V1-5GAG, and versikine was quantified by sandwich ELISA at different time points. The *solid lines* represent a nonlinear regression fit of the data as described in the Experimental procedures. Data are reported as mean ± SD (n = 3 independent experiments). IB, immunoblot; VSK, versikine.

ADAMTS9 MDTCS versicanase activity was initially assessed against purified versican V1 isoform, using both the full-length form and the V1-5GAG construct (truncated after F694 in the GAGβ domain) (47). Increasing concentrations of ADAMTS9 MDTCS (0.025–25 nM) were incubated with 100 nM of each substrate for 2 h, and after deglycosylation with chondroitinase ABC the cleavage products were analyzed by immunoblotting using anti-Vc, raised against the sequence ^432^TVPKDPEAAEARRG^445^ which spans the “canonical” versicanase cleavage site at E441-A442 (47), and a neoepitope antibody raised against the sequence ^436^DPEAAE^441^, which specifically detects the N-terminal product of this cleavage, versikine (48) (Fig. 4, *A* and *B*). Neoepitope antibodies do not recognize their epitope in the context of the intact protein and detect only the specific cleavage fragment (49). The anti-Vc immunoblots (Fig. 4, *C* and *D*) showed that bands corresponding to uncleaved V1 and V1-5GAG decreased in intensity in the presence of higher concentrations of ADAMTS9 MDTCS with maximal proteolysis observed in the presence of 25 nM enzyme over 24 h. Conversely, the ∼75 kDa band, corresponding to versikine in the anti-Vc immunoblots, was absent or faintly present at the lowest concentrations of ADAMTS9 MDTCS, increasing in intensity as the protease concentration rose. In comparison, 5 nM full length ADAMTS5, estimated to be the most potent versicanase (37), completely converted the two substrates into versikine (Fig. 4, *C* and *D*). We then tested inhibition of ADAMTS9 MDTCS versicanase activity by the four TIMPs. ADAMTS9 MDTCS (25 nM) was incubated with TIMP-1, TIMP-2, TIMP-3, or TIMP-4 (each at 500 nM) for 1 h before addition of V1-5GAG (100 nM, 2 h, 37 °C). Under these conditions, TIMP-3 completely inhibited versikine generation, while TIMP-2 showed only partial inhibition (Fig. 4*E*), thus confirming the choice of TIMP-3 for active site titration of ADAMTS9 MDTCS (Fig. 3*C*). The versicanase activity of ADAMTS9 MDTCS against V1 and V1-5GAG was also quantitatively assessed by a previously described sandwich ELISA which specifically measures versikine levels (37, 50) (Fig. 4*F*). ADAMTS9 MDTCS cleaved V1 and V1-5GAG with specificity constants (*k*_cat_/*K*_m_) of (2.1 ± 0.7) × 10^4^ and (1.9 ± 0.4) × 10^4^ M^−1^s^−1^ (n = 5), respectively.

### Identification of ADAMTS9 MDTCS cleavage sites in versican V1

**Figure 5:**
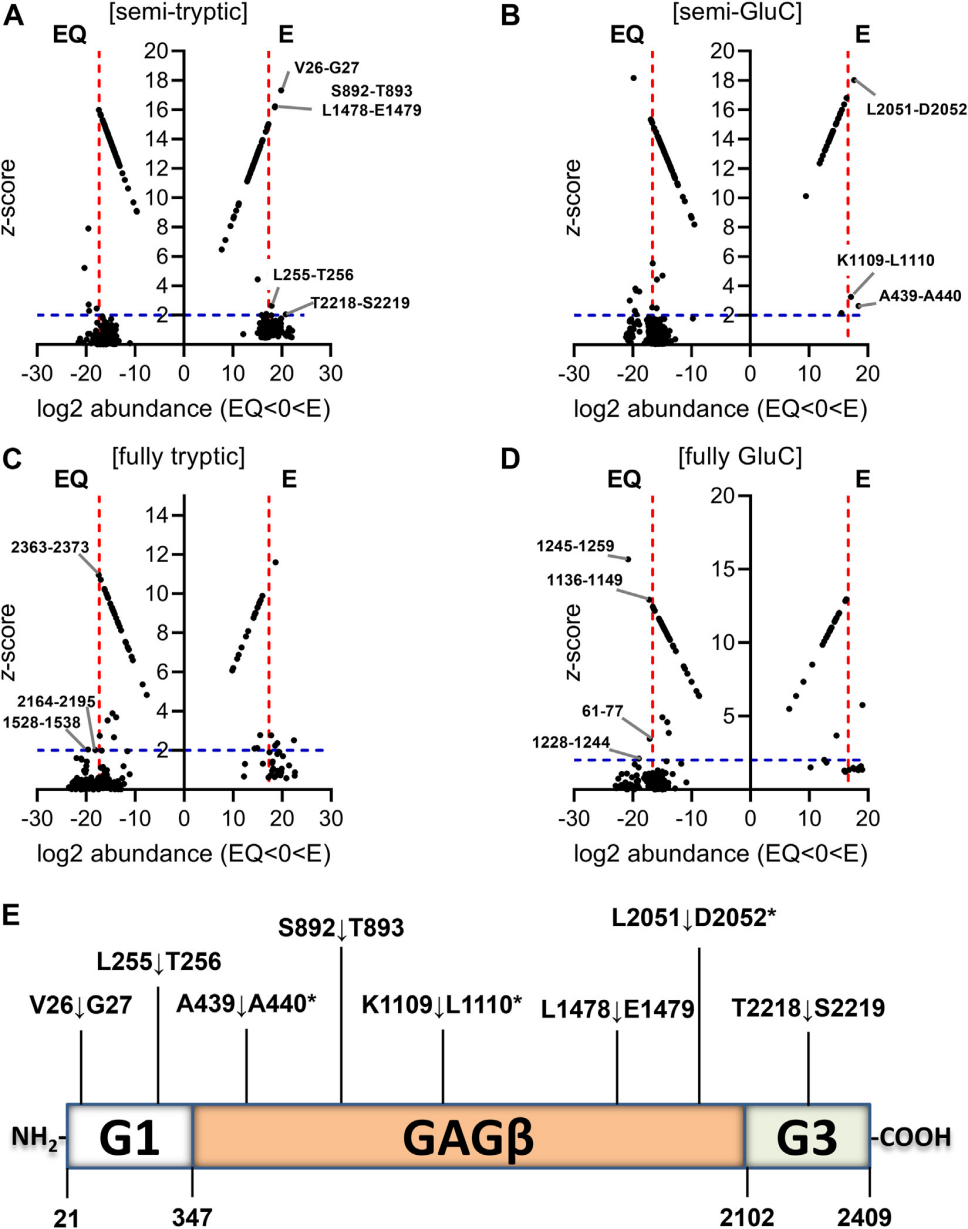
**Identification of ADAMTS9 MDTCS cleavage sites in versican V1.***A–D*, plots of log2 peptide abundance *versus z*-score for semi-tryptic (*A*), semi-GluC (*B*), fully tryptic (*C*), and fully GluC (*D*) peptides. *Dashed blue* and *red lines* indicate the thresholds for *z*-score and log2 peptide abundance, respectively. Cleavage sites originating from semi-tryptic (*A*), semi-GluC (*B*) peptides significantly more abundant in the E digests are indicated. In *panels C* and *D*, first and last amino acid of significant peptides more abundant in the EQ digests are indicated. *E*, overview of ADAMTS9 cleavage sites in versican V1. *Asterisks* indicate cleavage sites identified in digests treated with GluC. Domains are not drawn to scale.

ADAMTS9 MDTCS cleavage sites in versican V1 were identified using LFQ proteomics and *z*-score analysis (Fig. 5) as recently applied to identify ADAMTS cleavage sites in versican V1 (38), osteopontin (51), and cartilage oligomeric matrix protein (52). As part of this analysis, we also sought to determine whether digestion with GluC could improve detection of cleavage events in versican and aggrecan, given their scarcity of R/K residues and the abundance of E/D residues in the central GAG-rich regions, contrasting with other proteoglycans, such as biglycan (Figs. S2 and S3).

Five semi-tryptic and three semi-GluC peptides were significantly higher in ADAMTS9 E digests for a total of eight unique potential cleavage sites resulting from ADAMTS9 MDTCS activity, two in the G1 domain, five in the central GAGβ region, and one in the G3 domain (Fig. 5, *A*, *B*, and *E*, and Table 2). None of these were supported by higher abundance of fully tryptic/GluC peptides in the EQ digests (Fig. 5, *C* and *D*). Three fully tryptic peptides (residues 1528–1538, 2164–2195, and 2363–2373) and four fully GluC peptides (residues 61–77, 1136–1149, 1228–1244, and 1245–1259) were significantly more abundant in EQ digests, suggesting additional cleavage sites not identified by semi-tryptic or semi-GluC peptides (Fig. 5, *C* and *D*).

**Table 2:** Peptides with significant *z*-scores from ADAMTS9 MDTCS digests of versican V1

Semi-tryptic or semi-GluC peptide	Working protease	Peptide parameters			
		Cleavage site position	Domain	Log2 abundance	*Z*-score
**[V].G**KSPPVR.[G]	Tryp	V26-G27	G1	19.9	17.3
**[L].T**VPSKFTFEEAAK.[E]	Tryp	L255-T256	G1	2.64	17.9
**[A].A**EARRGQFE.[S]	GluC	A439-A440	GAGβ	2.65	18.4
**[S].**Ac**T**EPTGLVLSTVMDRVVAENITQTSR.[E]	Tryp	S892-T893	GAGβ	18.6	16.2
**[K].L**WSRQE.[V]	GluC	K1109-L1110	GAGβ	17.1	3.25
**[L].E**KHPEVPSAK.[A]	Tryp	L1478-E1479	GAGβ	18.7	16.2
[E].INP^†^ETQAALIRGQDSTIAASEQQVAARI**L.[D**]	GluC	L2051-D2052	GAGβ	18.0	17.7
[R].LQGAHL**T.[S]**	Tryp	T2218-S2219	G3	20.8	2.06

Residues within brackets precede or follow the detected peptide sequence (within full stops). P1 and P1′ residues are in bold. *Z*-score is the number of standard deviations from the mean E/EQ ratio of all peptides found in both groups. Tryp, trypsin. Residues were numbered according to UniProt ID P13611-2. “Ac” indicates amino acid acetylation. † indicates amino acid oxidation (P).

### Identification of cleavage sites in versican V2 by ADAMTS1, ADAMTS4, ADAMTS5, and ADAMTS9 MDTCS

**Figure 6:**
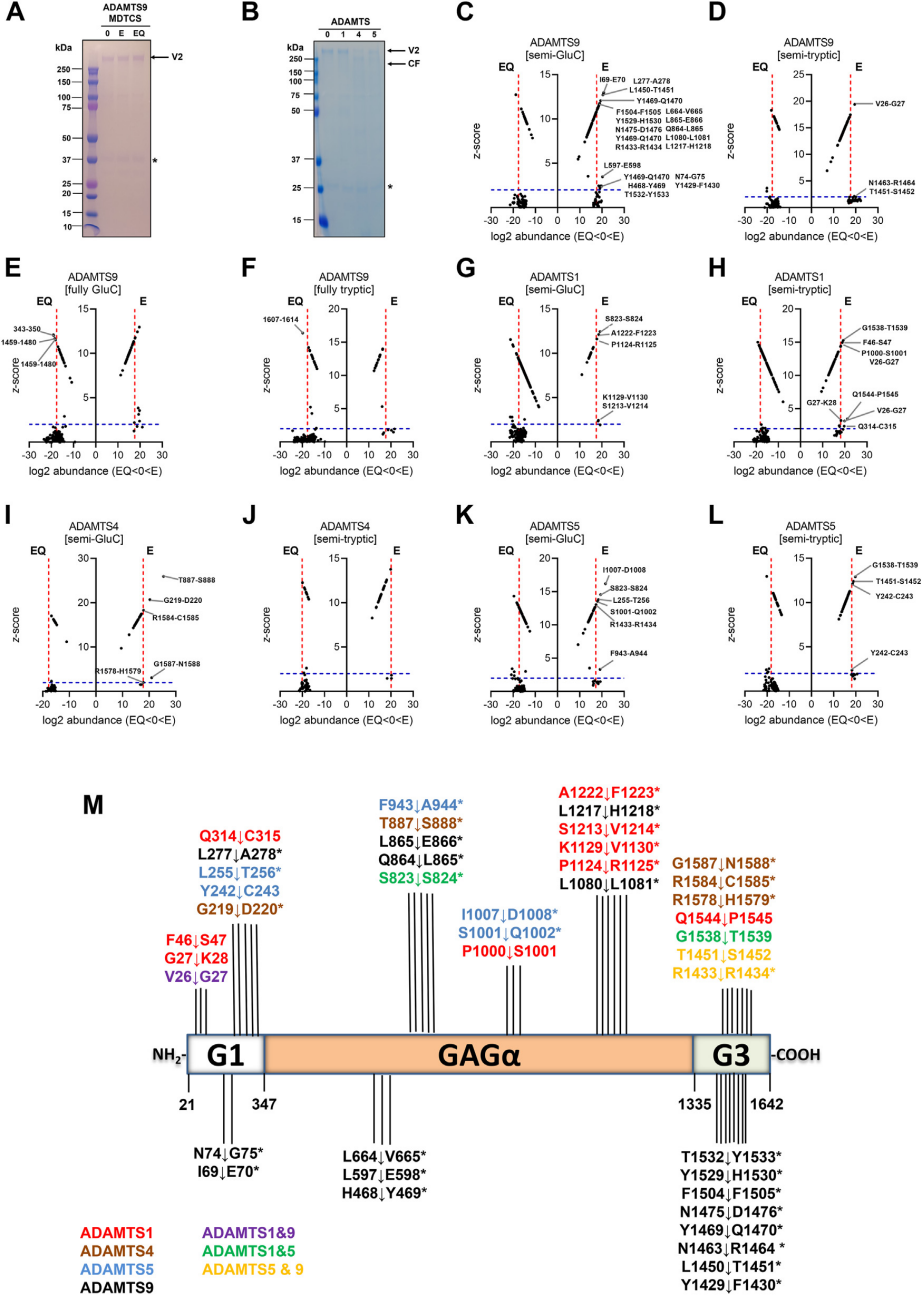
**Identification of ADAMTS9 MDTCS, ADAMTS1, ADAMTS4, and ADAMTS5 cleavage sites in versican V2.***A* and *B*, Coomassie blue staining of versican V2 (100 nM) digested with ADAMTS9 MDTCS E or EQ (25 nM, *A*), ADAMTS1, ADAMTS4, or ADAMTS5 (10 nM, *B*). *Asterisks* indicate bands detected also in the undigested controls (0). *C* and *D*, plots of log2 peptide abundance *versus z*-score for semi-GluC (*C*) and semi-tryptic (*D*) V2 peptides generated by ADAMTS9 MDTCS. Cleavage sites are indicated. *E* and *F*, fully GluC (*E*) and fully tryptic (*F*) peptides detected in ADAMTS9 MDTCS E and EQ digests. First and last amino acid of peptides significantly more abundant in the EQ digests are indicated. *Dashed blue* and *red lines* indicate the thresholds for *z*-score and log2 peptide abundance, respectively. *G–L*, plots of log2 peptide abundance *versus z*-score for semi-GluC (*G*, *I*, and *K*) and semi-tryptic (*H*, *J*, and *L*) V2 peptides generated by ADAMTS1 (*G* and *H*), ADAMTS4 (*I* and *J*), and ADAMTS5 (*K* and *L*). Cleavage sites are indicated. *Dashed blue* and *red lines* indicate the thresholds for *z*-score and log2 peptide abundance, respectively. *M*, overview of ADAMTS9 MDTCS, ADAMTS1, ADAMTS4, and ADAMTS5 cleavage sites in versican V2. *Asterisks* indicate cleavage sites identified in digests treated with GluC. Cleavage at N1463-R1464 was identified by one semi-tryptic and three fully GluC peptides. CF, cleavage fragments.

While our previous analysis (38) and the current study identified cleavage sites by ADAMTS1, ADAMTS4, ADAMTS5, and ADAMTS9 in the GAGβ region of versican, only one ADAMTS4 cleavage site in GAGα (E405-Q406) was previously reported (53). We aimed to identify additional ADAMTS cleavages by applying the LFQ proteomics and *z*-score analysis to the versican V2 isoform, which was transiently expressed in HEK293T cells and purified by diethylaminoethyl chromatography. Purified versican V2 was incubated separately with purified recombinant ADAMTS9 MDTCS E or EQ (25 nM each) for 24 h at 37 °C. As lack of a suitable antibody against the GAGα region precluded analysis by immunoblot, digest products were visualized by CBB staining (Fig. 6, *A* and *B*). No specific versican V2 cleavage bands were detected in the presence of ADAMTS9 MDTCS (Fig. 6*A*), whereas after digestion with full-length ADAMTS4 or ADAMTS5 (10 nM) for 2 h at 37 °C, the intensity of the ∼300 kDa band corresponding to full-length V2 decreased, with appearance of a ∼250 kDa band (Fig. 6*B*). Limited V2 proteolysis was observed upon incubation with full-length ADAMTS1 (Fig. 6*B*).

As proteoglycans stain poorly on CBB and fragments derived from cleavage events close to protein termini are difficult to detect, we further used the LFQ proteomics and *z*-score approach to identify V2 peptides arising from ADAMTS9 MDTCS activity. Two hundred and seventy-nine semi-tryptic, 160 fully tryptic, 303 semi-GluC, and 169 fully GluC versican V2 peptides were identified, covering 89% of the molecule (79% trypsin alone and an additional 10% with GluC digestion). Two-step stratification identified 21 cleavage sites, four in the G1 domain, seven in the GAGα domain, and 10 in the G3 domain (Fig. 6, *C*, *D*, and *M*, and Table 3). Cleavage at V26-G27 was also detected in V1 digests (Table 2). Cleavages at N1463-R1464, Y1469-Q1470, and N1475-D1476 were supported by fully GluC peptides significantly more abundant in the EQ control digests covering residues 1459 to 1480 (Fig. 6*E*). One fully GluC peptide (residues 343–350) and one fully tryptic peptide (residues 1607–1614) were significantly more abundant in ADAMTS9 EQ digests, suggesting additional cleavage sites not identified by semi-GluC or semi-tryptic peptides (Fig. 6, *E* and *F*). Cleavage at L1450-T1451 in the G3 domain corresponds to a cleavage site previously identified in ADAMTS5 digests of versican V1 (L2217-T2218) (38).

**Table 3:** Peptides with significant *z*-score from ADAMTS9 MDTCS digests of versican V2

Semi-tryptic or semi-GluC peptide	Corresponding spanning fully tryptic or fully GluC peptide sequence	Working protease	Semi-tryptic or semi-GluC peptide parameters				Corresponding spanning peptide		
			Cleavage site position	Domain	Log2 abundance	*Z*-score	Position	Log2 abundance	*Z*-score
**[V].G**KSPPVR.[G]		Tryp	V26-G27	G1	19.5	19.4			
**[I].E**VDKNGKDLKE.[T]		GluC	I69-E70	G1	18.2	11.2			
[E].FLRIKWSK**I.[E]**					20.7	12.9			
[E].FLRIKWSKIEVDK**N.[G]**		GluC	N74-G75	G1	19.1	2.29			
[E].AAKECENQDAR**L.[A]**		GluC	L277-A278	G1	18.0	11.2			
[E].VLQSTTGVS**H.[Y]**		GluC	H468-Y469	GAGα	19.5	2.46			
[E].VITVSKTSEDTIHTHLED**L.[E]**		GluC	L597-E598	GAGα	20.2	3.46			
[E].IELFPYSGDKI**L.[V]**		GluC	L664-V665	GAGα	18.5	11.5			
[E].LFPYSGDKI**L.[V]**					20.8	13.0			
[E].FTLIPDSTQK**Q.[L]**		GluC	Q864-L865	GAGα	18.9	11.8			
[E].FTLIPDSTQKQ**L.[E]**		GluC	L865-E866	GAGα	18.2	11.3			
**[L].L**TGSERVPVLE.[T]		GluC	L1080-L1081	GAGα	17.8	11.1			
[E].FSTIKVTVPSDITTAFSSVDR**L.[H]**		GluC	L1217-H1218	GAGα	18.8	11.7			
**[Y].F**AHRRTWDAAERE.[C]		GluC	Y1429-F1430	G3	19.1	2.19			
**[R].R**TWDAAERE.[C]		GluC	R1433-R1434	G3	18.0	11.2			
[E].C^#^RLQGAH**L.[T]**		GluC	L1450-T1451	G3	18.3	11.3			
[E].CRLQGAH**L.[T]**					20.4	12.7			
[R].LQGAHL**T.[S]**		Tryp	T1451-S1452	G3	20.1	2.00			
[R].LQGAHLTSILSHEEQMFV**N.[R]**		Tryp	N1463-R1464	G3	19.3	2.11			
	[E].QMFV**NR**VGHDYQWIGLNDKMFE.[H]	GluC					1459–1480	18.5∗	11.7
	[E].QM^†^FV**NR**VGHDYQWIGLNDKMFE.[H]						1459–1480	19.1∗	12.1
	[E].Q^#^M^†^FV**NR**VGHDYQWIGLNDKMFE.[H]						1459–1480	18.2∗	11.5
**[Y].Q**WIGLNDKM^†^FE.[H]	[E].QMFVNRVGHD**YQ**WIGLNDKMFE.[H]	GluC	Y1469-Q1470	G3	19.4	12.1	1459–1480	18.5∗	11.7
[E].Q^#^M^†^FVNRVGHD**Y.[Q]**	[E].QM^†^FVNRVGHD**YQ**WIGLNDKMFE.[H]				18.2	11.3	1459–1480	19.1∗	12.1
[E].Q^#^MFVNRVGHD**Y.[Q]**	[E].Q^#^M^†^FVNRVGHD**YQ**WIGLNDKMFE.[H]				18.5	2.46	1459–1480	18.2∗	11.5
[E].Q^#^MFVNRVGHDYQWIGL**N.[D]**	[E].QMFVNRVGHDYQWIGL**ND**KMFE.[H]	GluC	N1475-D1476	G3	17.9	11.1	1459–1480	18.5∗	11.7
[E].Q^#^M^†^FVNRVGHDYQWIGL**N.[D]**	[E].QM^†^FVNRVGHDYQWIGL**ND**KMFE.[H]				18.2	11.3	1459–1480	19.1∗	12.1
	[E].Q^#^M^†^FVNRVGHDYQWIGL**ND**KMFE.[H]						1459–1480	18.2∗	11.5
[E].NWRPNQPDS**F.[F]**		GluC	F1504-F1505	G3	18.9	11.8			
**[Y].H**LTYTCKKGTVACGQPPVVE.[N]		GluC	Y1529-H1530	G3	18.7	11.6			
[E].NGQWNDVPCNYHL**T.[Y]**		GluC	T1532-Y1533	G3	19.9	2.44			

Residues within brackets precede or follow the detected peptide sequence (within periods). P1 and P1′ residues are in bold. *Z*-score is the number of standard deviations from the mean E/EQ ratio of all peptides found in both groups. Tryp, trypsin. Residues are numbered according to UniProt ID P13611-3. Asterisk indicates peptide abundances that were higher in ADAMTS9 EQ digests. All other peptide abundance values were higher in the ADAMTS9 E digests. # indicates amino acid cyclization (Q, C), † indicates amino acid oxidation (M).

We then subjected ADAMTS1, ADAMTS4, and ADAMTS5 digests of V2 to LFQ proteomics and *z*-score analysis to identify additional cleavage sites by these proteases (Table 4 and Fig. 6, *G*–*L*). Of the 528 semi-GluC and the 382 semi-tryptic peptides identified in the ADAMTS1 digests of versican V2, five and eight were significantly more abundant in the E than in the EQ control digests, respectively, providing 12 new cleavage sites (Figs. 6, *G* and *H* and S4). The cleavage site at S1213-V1214 was additionally supported by a corresponding fully GluC spanning peptide (residues 1196–1215) significantly more abundant in the EQ digests (Fig. S4*A*). Three tryptic peptides (residues 120–155, 171–211, and 171–214) were significantly more abundant in the ADAMTS1 EQ digest (Fig. S4*B*), suggesting additional cleavage sites in the G1 domain.

**Table 4:** Peptides with significant *z*-scores from ADAMTS1, ADAMTS4, and ADAMTS5 digests of versican V2

Protease	Semi-tryptic or semi-GluC peptide	Corresponding spanning fully tryptic or fully GluC peptide sequence	Working protease	Semi-tryptic or semi-GluC peptide parameters				Corresponding spanning peptide		
				Cleavage site position	Domain	Log2 abundance	*Z*- score	Position	Log2 abundance	*Z-* score
ADAMTS1	**[V].G**KSPPVR.[G]		Tryp	V26-G27	G1	18.2	14.4			
	**[V].G**KSPPVRGSLSGK.[V]					19.9	3.2			
	**[G].K**SPPVRGSLSGK.[V]		Tryp	G27-K28	G1	18.3	3.22			
	[K].AcVSLPCH**F.[S]**		Tryp	F46-S47	G1	18.9	15.0			
	**[Q].C**GGGLLGVR.[T]		Tryp	Q314-C315	G1	19.7	2.37			
	[E].AATVSKWSWDEDNTTSKP^†^LESTEP^†^SA**S.[S]**		GluC	S823-S824	GAGα	19.1	12.4			
	**[P].S**QDILVIDQTR.[L]		Tryp	P1000-S1001	GAGα	18.2	14.4			
	**[P].R**IGPKVSLSPGPEQKYETE.[G]		GluC	P1124-R1125	GAGα	11.6	17.7			
	**[K].V**SLSPGPEQKYE.[T]		GluC	K1129-V1130	GAGα	18.3	2.34			
	[E].FSTIKVTVPSDITTAFS**S.[V]**	[E].FSTIKVTVPSDITTAFS**SV**D.[R]	GluC	S1213-V1214	GAGα	18.4	2.44	1196–1215	18.0∗	2.23
	[E].FSTIKVTVPSDITTAFSSVDRLHTTS**A.[F]**		GluC	A1222-F1223	GAGα	18.6	12.1			
	**[G].**Ac**T**VACGQP^†^PVVENAK.[T]		Tryp	G1538-T1539	G3	19.3	15.2			
	**[Q].P**PVVENAK.[T]		Tryp	Q1544-P1545	G3	21.2	3.47			
ADAMTS4	**[G].D**KMGKAGVRTYGFRSPQE.[T]		GluC	G219-D220	G1	20.3	20.7			
	**[T].**Ac**S**TGIAEKSTLRDSTTE.[E]		GluC	T887-S888	GAGα	25.4	26.0			
	[E].INSLIRYHCKDGFIQ**R.[H]**		GluC	R1578-H1579	G3	17.8	2.05			
	[E].INSLIRYHCKDGFIQRHLPTI**R.[C]**		GluC	R1584-C1585	G3	17.9	18.3			
	[E].INSLIRYHCKDGFIQRHLPTIRCL**G.[N]**		GluC	G1587-N1588	G3	20.9	3.09			
ADAMTS5	**[Y].C**YVDHLDGDVFHLTVPSKFTFEEAAKECENQDAR.[L]		Tryp	Y242-C243	G1	18.6	12.1			
	**[Y].C**YVDHLDGDVFHLTVPSK.[F]					18.3	2.38			
	**[L].T**VPSKFTFEEAAKE.[C]		GluC	L255-T256	G1	18.4	13.8			
	[E].AATVSKWSWDEDNTTSKP^†^LESTEP^†^SA**S.[S]**		GluC	S823-S824	GAGα	19.4	14.6			
	[E].VEDVDLSKPVSTVPQ**F.[A]**		GluC	F943-A944	GAGα	19.2	3.33			
	**[S].**Ac**Q**DILVIDQTRLE.[A]		GluC	S1001-Q1002	GAGα	18.1	13.5			
	[E].VLGEPSQDILV**I.[D]**		GluC	I1007-D1008	GAGα	21.6	16.2			
	**[R].R**TWDAAERE.[C]		GluC	R1433-R1434	G3	17.4	13.0			
	[R].LQGAHL**T.[S]**		Tryp	T1451-S1452	G3	19.0	12.4			
	**[G].**Ac**T**VACGQP^†^PVVENAK.[T]		Tryp	G1538-T1539	G3	19.8	12.9			

Residues within brackets precede or follow the detected peptide sequence (within periods). P1 and P1′ residues are in bold. *Z*-score is the number of standard deviations from the mean E/EQ ratio of all peptides found in both groups. Tryp, trypsin. Residues are numbered according to UniProt ID P13611-3. Asterisks indicate peptide abundances that were higher in EQ digests. All other peptide abundance values were higher with the active constructs. “Ac” indicates amino acid acetylation, † indicates amino acid oxidation (P).

Of the 98 semi-GluC peptides identified in the ADAMTS4 digests of versican V2, five were significantly more abundant in the E than in the EQ digests, indicating five distinct cleavage sites (Fig. 6*I*). None of the 82 semi-tryptic peptides identified in the ADAMTS4 digests were significantly more abundant in the E than in the EQ digests (Fig. 6*J*). One fully tryptic peptide (residues 1573–1584) was more abundant in the EQ digests (Fig. S4*D*), suggesting potential cleavage sites in the G3 domain not supported by either semi-GluC or semi-tryptic peptides.

Of the 224 semi-tryptic and the 306 semi-GluC peptides identified in the ADAMTS5 digests of versican V2, four and six were significantly more abundant in the active than in the control digests, respectively, for a total of nine newly identified cleavage sites (Fig. 6, *K* and *L*). Four fully GluC (residues 275–282, 542–556, 557–568, 560–568) (Fig. S4*E*) and three fully tryptic (residues 215–224, 1612–1620, and 1621–1632) (Fig. S4*F*) peptides provided evidence for potential cleavage sites in the G1, GAGα, and G3 domains.

One cleavage site, V26-G27 in the G1 domain, was shared by ADAMTS1 and ADAMTS9 MDTCS, two sites (S823-S824 in the GAGα domain and G1538-T1539 in the G3 domain) were shared by ADAMTS1 and ADAMTS5, and two sites (R1433-R1434 and T1451-S1452 in the G3 domain) were shared by ADAMTS5 and ADAMTS9 (Fig. 6*M*). ADAMTS9 cleavage at T1451-S1452 in the G3 domain was detected at the corresponding site in digests of the V1 isoform (T2218-S2219) (Fig. 5*E* and Table 2).

### Aggrecanase activity of ADAMTS9 MDTCS

**Figure 7:**
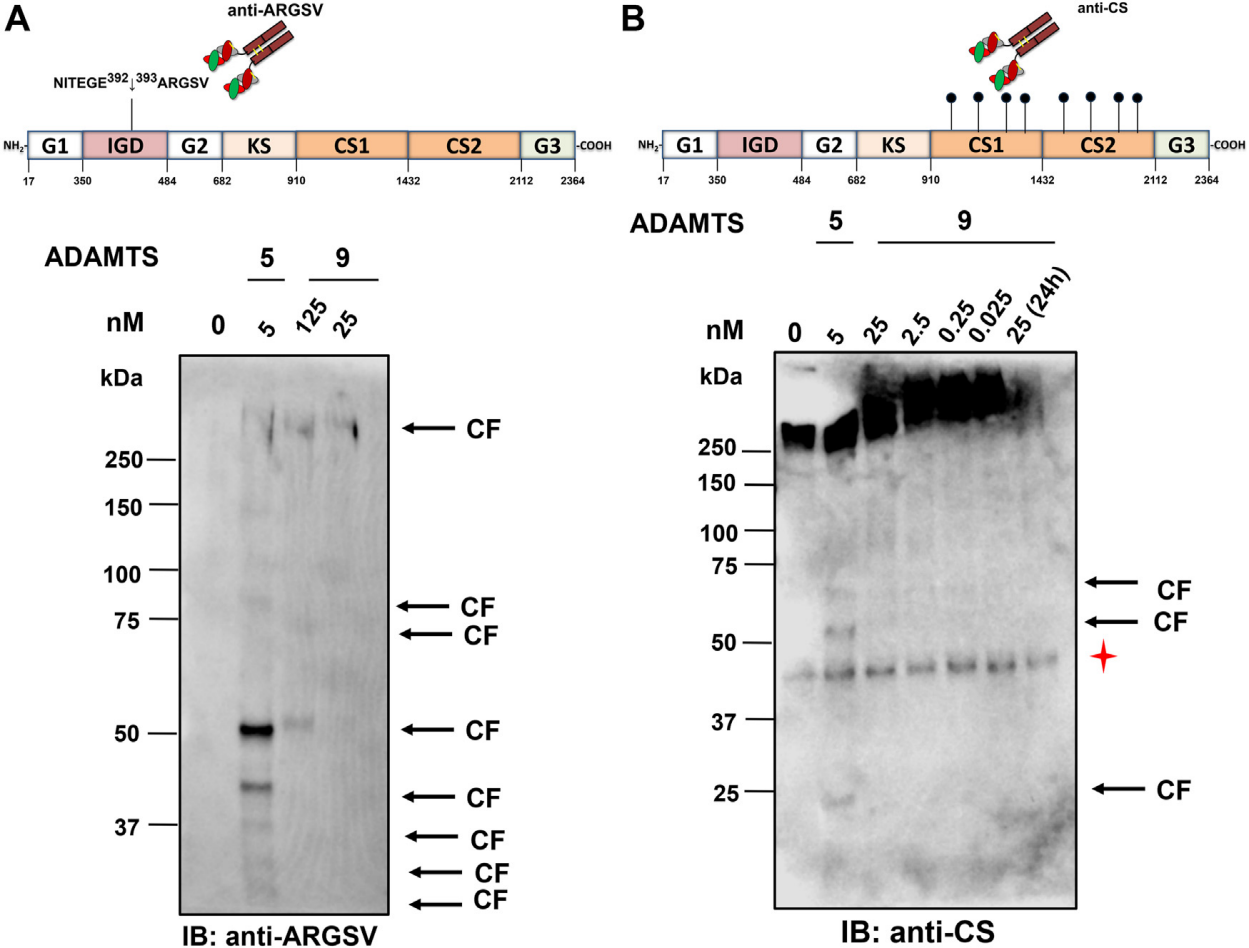
**Aggrecanase activity of ADAMTS9 MDTCS.** Increasing concentrations of ADAMTS9 MDTCS (0.025–25 nM) or 5 nM full-length ADAMTS5 were incubated with bovine aggrecan (400 nM). Digests were stopped after two or 24 h and cleavage fragments deglycosylated and subjected to SDS-PAGE. Bands were detected using anti-aggrecan ARGSV neoepitope (*A*) or CS-GAG stubs left following chondroitinase ABC treatment (*B*). The epitopes recognized by the antibodies are shown on the schematic of aggrecan. “0” condition refers to buffer control. In *B*, the *red asterisk* indicates a ubiquitous band. Numbering is according to bovine aggrecan, UniProt ID P16112-1. Domains are not drawn to scale. CF, cleavage fragment; CS, chondroitin sulfate; IGD, interglobular domain; KS, keratan sulfate-rich region.

Historically, aggrecanase activity was defined as the ability to cleave aggrecan at the E392-A393 bond (corresponding to E373-A374 in the secreted proteoglycan, UniProt ID: P16112) in the interglobular domain, designating ADAMTS4 as aggrecanase-1 (54) and ADAMTS5 as aggrecanase-2 (55). We tested whether ADAMTS9 MDTCS cleaved bovine aggrecan (UniProt ID: P13608) at this site using a neoepitope antibody directed against the C-terminal fragment (anti-ARGSV) (Fig. 7*A*). The most potent aggrecanase at the E392-A393 site, ADAMTS5 (33, 34), was used for comparison. A >250 kDa band typical of aggrecanase-cleaved aggrecan, likely resulting from cleavage at both the E392-A393 and E1499-G1500 sites (49) and matching in size the highest molecular weight band generated by ADAMTS5, was observed after 24 h digestion with 25 to 125 nM ADAMTS9 MDCTS (Fig. 7*A*). Both proteases generated a second band of ∼50 kDa. ADAMTS5 generated additional low molecular weight anti-ARGSV reactive bands at 5 nM, as a result of further processing of aggrecan. None of these bands were present in undigested aggrecan. To test whether ADAMTS9 MDTCS cleaved aggrecan at additional cleavage sites, ADAMTS9 MDTCS was incubated with bovine aggrecan for 2 h and the digestion products were analyzed by immunoblotting with an antibody which recognizes the CS stubs left by chondroitinase ABC. Aggrecan was present as a major band with molecular weight >250 kDa (Fig. 7*B*). A band of approximately 40 kDa was detected in all samples analyzed, including undigested aggrecan, suggesting occurrence of limited proteolysis in the commercial aggrecan batch or cross-reaction of the antibody with a contaminant (Fig. 7*B*). Whereas ADAMTS5 (5 nM) generated three distinct cleavage fragments after 2 h incubation, these were not detected even after 24 h incubation with 25 nM ADAMTS9 MDTCS. Since this data did not exclude the possibility that ADAMTS9 generated alternative aggrecan fragments that were not detected by the anti-CS antibody, we considered using LFQ proteomics and *z*-score analysis to identify additional cleavage fragments. Unfortunately, peptide coverage of only 29% of aggrecan, which was primarily in the G1 and G2 domains, with little to no coverage in the CS-and G3 domains, limited identification of a sufficient number of semi-tryptic or semi-GluC peptides to determine cleavage events in any of these regions (Fig. S5*A*). Following deglycosylation with chondroitinase ABC and keratanase the number of peptides identified increased from 247 to 384 but coverage improved marginally to 31% with only a single additional peptide identified in the CS1 domain (Fig. S5*B*). The average gap between tryptic cleavage sites in the CS domains is 42 residues with the largest gap of 180 residues (Fig. S3), resulting in minimal fragmentation. In the CS1 domain alone, which is 678 amino acids in length, there are only 7 R/K residues (Fig. S3). In comparison, the average gap between GluC sites in the CS domains is 6.2 residues (Fig. S3). The high density of GAG-attachment sites very likely also resulted in fewer LC-MS/MS peptide matches, owing to variable mass modification by glycosylation. This compounded the paucity of arginine and lysine residues (Fig. S2) and would likely be a factor in the relatively low yield of GluC peptides.

### Identification of cleavage sites in ADAMTS9 MDTCS, ADAMTS1, ADAMTS4, and ADAMTS5 digests of biglycan

**Figure 8:**
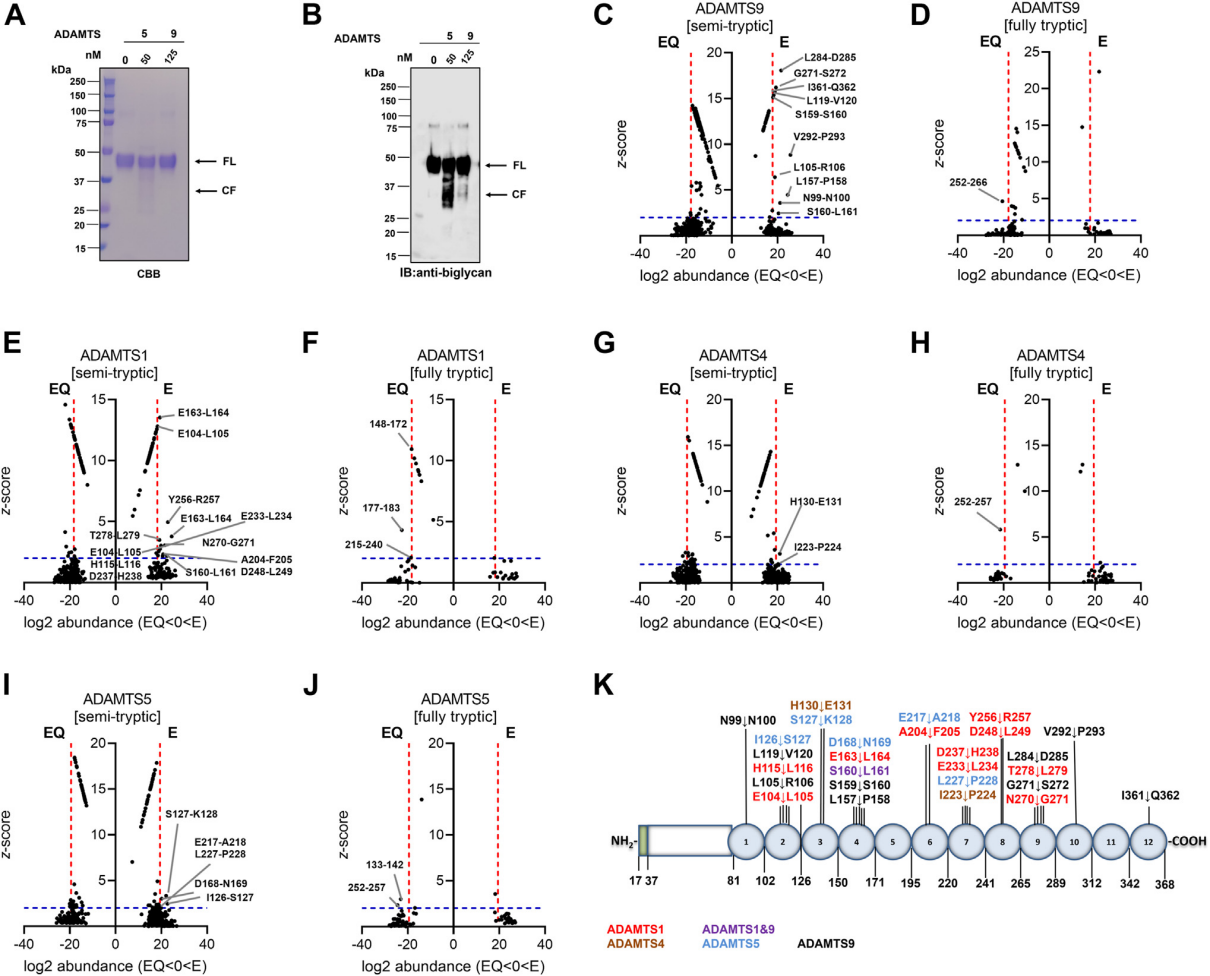
**ADAMTS9 MDTCS proteolytic activity against biglycan**. Activity of ADAMTS9 MDTCS (125 nM) and full-length ADAMTS5 (50 nM) against biglycan (2 μM) was investigated after 24 h digestion and deglycosylation. Coomassie Brilliant Blue (CBB) staining (*A*) and immunoblotting (*B*), using polyclonal anti-biglycan are shown after reducing SDS-PAGE. *C*–*J*, Plot of log2 peptide abundance *versus* z-score for semi-tryptic (*C*, *E*, *G*, and *I*) and fully tryptic (*D*, *F*, *H*, and *J*) biglycan peptides generated by ADAMTS9 MDTCS (*C* and *D*), ADAMTS1 (*E* and *F*), ADAMTS4 (*G* and *H*), and ADAMTS5 (*I* and *J*). In plots of semi-tryptic peptide abundance, cleavage sites are indicated. In plots of fully tryptic peptide abundance, first and last amino acid of tryptic peptides significantly more abundant in the EQ digests are indicated. *Dashed blue* and *red lines* indicate the thresholds for z-score and log2 peptide abundance, respectively. *K*, overview of ADAMTS9 MDTCS, ADAMTS1, ADAMTS4, and ADAMTS5 cleavage sites in biglycan. Leucine-rich repeats are indicated by numbers inside the schematic. CF, cleavage fragments; FL, full-length.

We defined the proteolytic activity of ADAMTS9 MDCTS against the small leucine-rich proteoglycan biglycan, which functions both as a structural component and as an inflammatory signaling molecule (56). Biglycan has a protein core of 12 leucine-rich repeats (LRR) and CS-GAGs attached to S42 and S47 (UniProt ID: P21810) (57). We have previously shown that ADAMTS4 and ADAMTS5 have the highest biglycanase activity, followed by ADAMTS1 (51). We then compared ADAMTS9 MDTCS biglycanase activity (125 nM) with that of ADAMTS5 (50 nM). The 24 h-digests were deglycosylated with chondroitinase ABC and analyzed through SDS-PAGE followed by CBB staining and immunoblotting with a polyclonal antibody. Cleavage fragments between 30 and 40 kDa were generated by both proteases, although ADAMTS5 biglycanase activity was clearly more robust than that of ADAMTS9 MDTCS (Fig. 8, *A* and *B*).

We then subjected ADAMTS1, ADAMTS4, ADAMTS5 and ADAMTS9 MDTCS digests of biglycan to LFQ proteomics and *z*-score analysis to identify cleavage sites (Table 5 and Fig. 8, *C*–*K*). In biglycan, the abundance of trypsin cleavage sites is identical to the rest of the human proteome (Fig. S2) and the average gap between the sites is ∼9 residues (Fig. S3), thus resulting in 96% sequence coverage upon trypsin treatment for sample preparation.

**Table 5:** Peptides with significant z-scores from ADAMTS1, ADAMTS4, ADAMTS5, and ADAMTS9 MDTCS digests of biglycan

Protease	Semi-tryptic peptide	Corresponding spanning fully tryptic peptide sequence	Semi-tryptic peptide parameters				Corresponding spanning peptide		
			Cleavage site position	Domain	Log2 abundance	*Z*- score	Position	Log2 abundance	*Z*- score
ADAMTS9	**[N].N**DISELRK.[D]		N99-N100	LRR1	21.1	3.60			
	[K].EISPDTTLLDLQNNDISE**L.[R]**		L105-R106	LRR2	18.8	6.42			
	[K].GLQHLYA**L.[V]**		L119-V120	LRR2	18.3	15.4			
	**[L].P**SSLVELR.[I]		L157-P158	LRR4	24.4	4.47			
	[K].NHLVEIPPNLP**S.[S]**		S159-S160	LRR4	18.0	15.2			
	[K].NHLVEIPPNLPS**S.[L]**		S160-L161	LRR4	20.4	2.45			
	**[G].S**LSFLPTLR.[E]		G271-S272	LRR9	19.3	16.2			
	**[L].D**NNKLAR.[V]		L284-D285	LRR9	21.6	18.1			
	**[V].P**SGLPDLK.[L]		V292-P293	LRR10	25.6	8.84			
	**[I].Q**FGNYK.[K]		I361-Q362	LRR12	18.8	15.7			
ADAMTS1	[K].SVPKEISPDTTLLDLQNNDIS**E.[L]**		E104-L105	LRR2	18.5	2.22			
	[K].EISPDTTLLDLQNNDIS**E.[L]**				18.3	12.8			
	[K].DDFKGLQ**H.[L]**		H115-L116	LRR2	18.8	2.55			
	[K].NHLVEIPPNLPS**S.[L]**		S160-L161	LRR4	22.4	2.18			
	[K].NHLVEIPPNLPSSLV**E.[L]**	[K].NHLVEIPPNLPSSLV**EL**RIHDNRIR.[K]	E163-L164	LRR4	24.5	3.78	148–172	18.3∗	10.9
	**[E].L**RIHDNR.[I]				19.4	13.5			
	**[A].F**DGLKLNYLR.[I]		A204-F205	LRR6	20.5	2.18			
	**[E].L**HLDHNKIQAIELEDLLR.[Y]	[R].ISEAKLTGIPKDLPETLN**EL**HLDHNK.[I]	E233-L234	LRR7	21.5	3.06	215–240	18.6∗	2.07
	[K].DLPETLNELHL**D.[H]**		D237-H238	LRR7	19.4	2.76			
	[K].DLPETLNELHLDHNKIQAIELE**D.[L]**		D248-L249	LRR8	20.8	2.32			
	**[Y].R**LGLGHNQIR.[M]		Y256-R257	LRR8	22.8	4.95			
	**[N].G**SLSFLPTLR.[E]		N270-G271	LRR9	19.9	3.03			
	**[T].L**RELHLDNNK.[L]		T278-L279	LRR9	19.2	3.50			
ADAMTS4	**[H].E**KAFSPLR.[K]		H130-E131	LRR3	21.1	3.17			
	**[I].P**KDLPETLNELHLDHNK.[I]		I223-P224	LRR7	20.3	2.06			
ADAMTS5	**[I].S**KIHEKAFSPLR.[K]		I126-S127	LRR2/LRR3	22.7	2.45			
	**[S].K**IHEKAFSPLR.[K]		S127-K128	LRR3	22.1	3.35			
	[K].LYISKNHLVEIPPNLPSSLVELRIH**D.[N]**		D168-N169	LRR4	20.2	3.02			
	**[E].A**KLTGIPK.[D]		E217-A218	LRR6	19.5	2.19			
	**[L].P**ETLNELHLDHNKIQAIELEDLLRYSK.[L]		L227-P228	LRR7	19.9	2.00			

Residues within brackets precede or follow the detected peptide sequence (within periods). P1 and P1′ residues are in bold. *Z*-score is the number of standard deviations from the mean E/EQ ratio of all peptides found in both groups. Residues are numbered according to UniProt ID P21810. LRR, leucine-rich repeat. Asterisks indicate peptide abundances that were higher in EQ digests. All other peptide abundance values were higher with the active constructs.

Of the 414 semi-tryptic peptides identified in the ADAMTS9 MDTCS digests of biglycan, 10 were significantly more abundant in the E than in the EQ digests, indicating 10 cleavage sites (Fig. 8*C*). A fully tryptic peptide (residues 252–266) was significantly more abundant in the EQ digests (Fig. 8*D*), indicating additional cleavages in LRR8.

Of the 441 semi-tryptic peptides identified in the ADAMTS1 digests of biglycan, 13 were significantly more abundant in the E than in the EQ digests, indicating 11 cleavage sites (Fig. 8*E*). Cleavage at E104-L105 was identified by two semi-tryptic peptides on the P1 side of the scissile bond, while cleavage at E163-L164 was supported by two significant semi-tryptic peptides flanking the scissile bond and a tryptic peptide spanning residues 148 to 172 that was significantly more abundant in the EQ digests (Table 5 and Fig. 8*F*). Cleavage at E233-L234 was supported by a semi-tryptic peptide significantly more abundant in the E digests and a tryptic peptide spanning residues 215 to 240 significantly more abundant in the EQ digests (Table 5 and Fig. 8*F*).

Of the 619 semi-tryptic peptides identified in the ADAMTS4 digests of biglycan, two were significantly more abundant in the E than in the EQ digests, indicating two cleavage sites (Fig. 8*G*). A tryptic peptide spanning residues 252 to 257 was significantly more abundant in the ADAMTS4 EQ than in the ADAMTS4 E digests, suggesting additional cleavage site(s) not supported by semi-tryptic peptides more abundant in the ADAMTS4 E digests (Fig. 8*H*).

Of the 582 semi-tryptic peptides identified in the ADAMTS5 digests of biglycan, five were significantly more abundant in the E than in the EQ digests, indicating five cleavage sites (Fig. 8*I*). Tryptic peptides spanning residues 133 to 142 and 252 to 257 were also significantly more abundant in the EQ than in the E digests, suggesting additional cleavage sites in the LRRs 3, five and 6 (Fig. 8*J*).

Overall, 27 novel cleavage sites by ADAMTS1, ADAMTS4, ADAMTS5, and ADAMTS9 MDTCS were identified in biglycan, one (S160-L161) being shared by ADAMTS1 and ADAMTS9 (Fig. 8*K*).

## Discussion

To address challenges in ADAMTS9 expression and purification (2, 14, 30, 31), we characterized a truncated construct (ADAMTS9 MDTCS, residues 1–877) consisting of all the “core domains” that were previously shown as essential for proteoglycanase activity in ADAMTS1 (35), ADAMTS4, and ADAMTS5 (33, 34, 37). We established a QF peptide cleavage assay based on an ADAMTS4 peptide substrate (44) for accurate determination of ADAMTS9 MDTCS concentrations by active site titration. Expression of ADAMTS9, ADAMTS1, ADAMTS4, and ADAMTS5 in the same cell system, application of the same purification protocol (58), and quantification of protein concentration by active-site titration (59), allowed us to directly compare the proteoglycanase activity of these proteases.

ADAMTS9 is unusual in being proteolytically active in its zymogen form, with prodomain removal by furin found to reduce its versicanase activity in an earlier study (32). As our ADAMTS9 MDTCS construct was not engineered to abrogate the furin cleavage sites at R74, R209 and R287, our preparations were fully furin-processed, as shown by SDS-PAGE/CBB staining. Since furin is ubiquitously expressed (60) and other proprotein convertases can cleave at multibasic recognition sites (61), it is likely that a high proportion of secreted ADAMTS9 undergoes prodomain removal *in vivo*, as observed in HEK29 T cells in this study. Fragmentation of HEK293- and COS-1-expressed ADAMTS9 was previously reported (2, 32, 62) but was not observed in our catalytically incompetent EQ construct, suggesting autolysis. The *z*-score approach correspondingly identified five putative autolytic sites, four of which were in the Sp domain. Autolytic C-terminal processing has been reported before for ADAMTS1 (35, 63, 64), ADAMTS4 (65), ADAMTS7 (66), ADAMTS8 (51), and ADAMTS17 (67), and may represent a crucial regulatory post-translational mechanism in ADAMTS proteases (68) as the C-terminal domains are known to contain distally located substrate-binding sites (exosites) required for proteolytic activity against native substrates (42). The Sp domain is indeed a major hotspot for exosites in ADAMTS1 (35), ADAMTS4, and ADAMTS5 (37). In addition to four autolytic sites in the Sp domain of ADAMTS9 MDCTS, we identified one in the prodomain (A85-F86), 11 residues downstream of a known furin processing site (R74). More studies are required to ascertain the biological significance of these autolytic events.

Here, we focused on versican processing owing to the strong evidence for versican being a critical ADAMTS9 substrate. ADAMTS9 MDTCS versicanase activity, determined using sandwich ELISA, was approximately 165-fold lower than ADAMTS5, 9-fold lower than ADAMTS4 (37), and 6-fold higher than ADAMTS1 (35), but this may have been underestimated in our assays. Moreover, although the C-terminal TSRs in ADAMTS1 and ADAMTS5 do not contribute to versicanase activity (35, 37), we cannot exclude that in ADAMTS9 the ancillary domains C-terminal to the Sp are also involved in versican binding and proteolysis. In particular, the function of the GON-1 domain remains largely unknown. A yeast two-hybrid screen using ADAMTS9 GON-1 domain as a bait identified low-density lipoprotein receptor-related protein-1 (LRP1) as a binding partner (19), suggesting that GON-1 could mediate ADAMTS9 LRP1-dependent endocytosis. Given the difficulties in expressing full-length ADAMTS9, an indirect way to test a possible involvement of GON-1 in versicanase activity may be carrying out direct binding assays of the isolated GON-1 domain to versican.

Underestimation of ADAMTS9 MDTCS versicanase activity may be also intrinsic to the assay we used to quantify it. The sandwich ELISA used in this study involves capture of versikine by a neoepitope antibody followed by recognition with an anti-G1 domain antibody (50). We did not identify any semi-tryptic peptides derived from cleavage at E441-A442 in our *z*-score analysis of the V1 isoform digested with ADAMTS9, while semi-tryptic peptides comprising the DPEAAE neoepitope were abundant in our previous analysis of ADAMTS1, ADAMTS4, and ADAMTS5 digests (38). This may be explained by the presence of the cleavage event at A439-A440 which will generate downstream semi-tryptic peptides too short to be detected by LC-MS/MS after ADAMTS9 MDTCS cleavage at E441-A442, which immunoblot and versikine ELISA demonstrated. Prior work suggests that this cleavage will likely disrupt the neoepitope recognized by anti-DPEAAE (47), leading to underestimation of ADAMTS9 MDTCS versicanase activity. Two cleavage sites in the G1 domain were also significant and these may further interfere with recognition by the detecting antibody.

In contrast to the versican GAGβ domain, prior knowledge of ADAMTS cleavage sites in the GAGα domain, present in versican V0 and V1 isoforms (28), was limited. We did not directly analyze cleavage sites in versican V0, as expression of this isoform is challenging due to its large size (372 kDa *versus* 265 and 182 kDa for V1 and V2, respectively) and correspondingly higher number of GAGs (17–23 *versus* 12–15 for V1 and 5–8 for V2) (69). Instead, we used the V2 isoform to characterize cleavage sites in the GAGα region of versican. This isoform is highly expressed in the central nervous system (70, 71), but it is also detected in vascular beds (72). We then applied LFQ proteomics and *z*-score analysis to carry out a comprehensive characterization of cleavage sites by ADAMTS1, ADAMTS4, ADAMTS5, and ADAMTS9 MDTCS on the versican V2 isoform. By combining data from trypsin and GluC digestions, we identified 9 ADAMTS1-, 5 ADAMTS4-, 5 ADAMTS5-, and 18 ADAMTS9- specific cleavage sites in V2. In addition, we identified one cleavage site shared by ADAMTS1 and 9, two cleavage sites shared by ADAMTS1 and 5, and two cleavage sites shared by ADAMTS5 and 9, for a total of 42 novel cleavage sites in V2, 17 of which in the GAGα domain. An ADAMTS4 cleavage site at E405-Q406 was identified by immunoblots with neoepitope antibodies on human brain tissue extracts (53), while ADAMTS1 cleavage at E950-G951 was identified using synthetic versican peptides 40 to 42 amino acid-long instead of full-length V2 (73). A semi-GluC peptide corresponding to the E405-Q406 was found exclusively in ADAMTS9 E digests of versican V2 in our analysis but was not statistically significant. Limitations intrinsic to the mass-spectrometry approach, for example a low density of tryptic sites in V2 (Fig. S3) may have hampered identification of these sites.

The relevance of the non-canonical cleavage sites identified in this and in our previous study (38) is supported by the versican turnover observed in two different mouse strains genetically engineered to express a mutant versican resistant to ADAMTS proteolysis at the canonical E441-A442 site (74, 75).

Because of the major role of ADAMTS5 in aggrecan turnover, and the prior reports of cooperative roles of ADAMTS5 and ADAMTS9 in versican turnover, we also examined aggrecan cleavage by ADAMTS9. We found that ADAMTS9 MDTCS has low aggrecanase activity, consistent with previous findings by Somerville *et al.* (2) and Zeng *et al.* (30). Somerville *et al.* incubated ADAMTS9-transfected HEK293 cells with bovine aggrecan and observed poor cleavage at E1790-A1791 (corresponding to E1953-A1954 in human aggrecan, UniProt ID: P16112) using antibodies against the AGEG neopeptide and comparing with ADAMTS4-transfected cells (2). Poor aggrecanase activity at the E1790-A1791 and E392-A393 sites was confirmed by Zeng *et al.* using an ADAMTS9 construct truncated after the central TSR (F649), *i.e.*, much shorter than the construct used in the present study (30), in agreement with our data. Rogerson *et al.* identified a third aggrecanase activity with the distinct ability to cleave at E-G sites (but not at the E392-A393 or E1953-A1954 sites, human aggrecan numbering, UniProt ID P16112) in the cartilage of *Adamts4*/*Adamts5* double knockout mice (76). Based on the specific up-regulation by retinoic acid at both mRNA and protein level, they speculated that ADAMTS9 could be the protease responsible for residual aggrecanase activity present in *Adamts4*/*Adamts5* double knockout mice (22). Our study does not support this possibility. However, as ADAMTS9 is active in its zymogen form prior to extracellular secretion (32), ADAMTS9 intracellular aggrecanase activity may be relevant in the secretory pathway, for example, during differentiation of progenitor stromal cells (77).

Compared to the rest of the proteome, aggrecan (both human and bovine) has a much higher frequency of glutamic acid residues (11% *versus* 7%), which, together with a lower frequency of lysine residues (1% *versus* 6%), and extensive and variable glycosylation, makes it very challenging to detect aggrecan peptides in LC-MS/MS.

Our LFQ proteomics and *z*-score analysis identified 27 novel cleavage sites generated by ADAMTS1, ADAMTS4, ADAMTS5, and ADAMTS9 MDCTS in biglycan. ADAMTS9 was reported to cleave mouse biglycan at L95-L96 in a terminal amine isotopic labeling of substrates (TAILS) experiment using tail skin from *Adamts9* conditional knockout mice *versus* wild-type littermates (27), but we did not find evidence of cleavage at the orthologous site in human biglycan.

Both ADAMTS4 and ADAMTS5 were shown to cleave biglycan at N186-C187 (78). In our *z*-score analysis, we found evidence for a semi-tryptic peptide (^177^GVFSGLRNMN^186^) in the ADAMTS5 digests which were more abundant in the active protease than in the control, but this was not significant.

In addition to ADAMTSs, proteases from different families also cleave biglycan. During biglycan synthesis, the astacin-like metalloprotease bone morphogenetic protein-1 (BMP-1), as well as the related mammalian Tolloid and mammalian Tolloid-like 1 (mTLL-1) proteases remove the N-terminal propeptide (79). BMP-1 cleaves biglycan at N37-D38 (79, 80). Matrix metalloprotease (MMP)3, MMP9, and MMP13 cleave biglycan at E104-L105 (81, 82), a site shared by ADAMTS1 in our analysis, while only MMP13 cleaves at G177-V178 (83). Granzyme-B cleaves biglycan at D91-T92 (84), while HtrA1 cleaves at eight sites (85), none of which were detected in our analysis. Thus, biglycan appears to be very susceptible to proteolytic attack, including by ADAMTS9.

Other than the proteoglycans analyzed in the present study, TAILS experiments have uncovered several ADAMTS9 ECM and transmembrane substrates such as MT1-MMP and Tmem67 (86), collagen α-1(XII), fibrillin-1, fibronectin (31), and periostin (27) which may also be relevant to the diverse phenotypes observed in *Adamts9* null/haploinsufficient mice. In particular, surface plasmon resonance experiments confirmed direct binding of ADAMTS9 and fibronectin, and fibronectin accumulation was observed in *Adamts9* knockout mouse embryos, strongly suggesting fibronectin as a physiologically relevant ADAMTS9 substrate (31).

**Figure 9:**
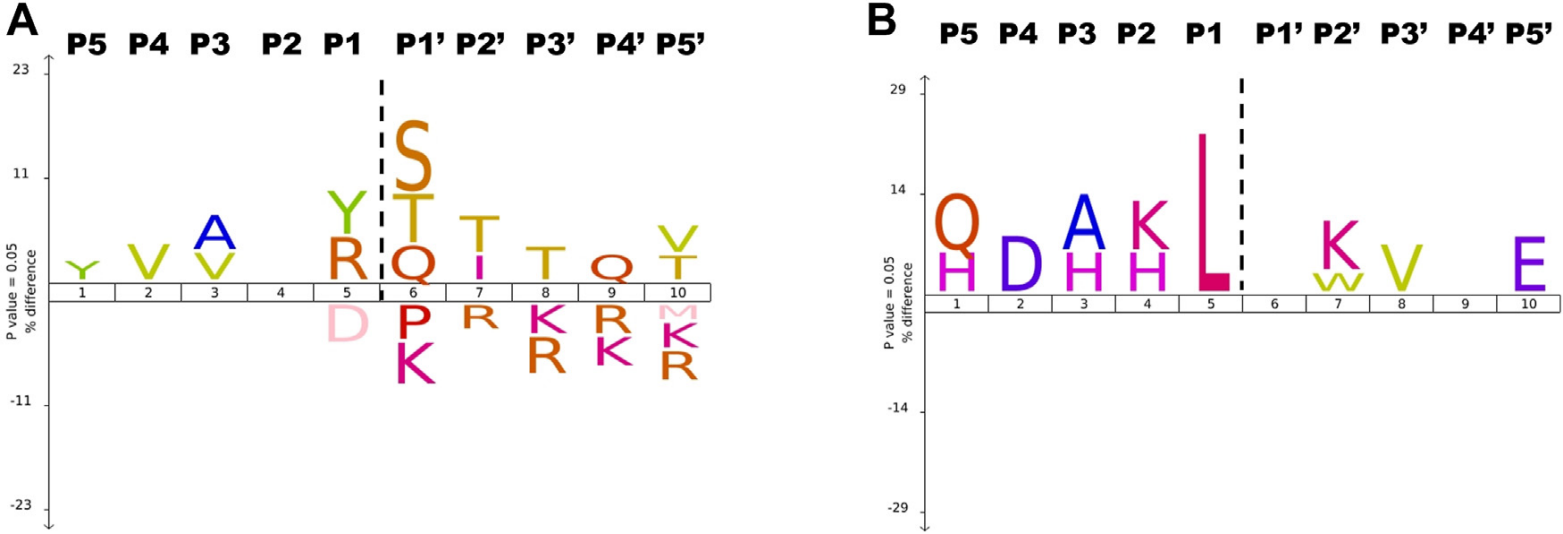
**IceLogo analysis of the cleavage site specificities of ADAMTS9 MDCTS for ECM substrates.** IceLogo analysis for all ECM substrates including proteoglycans (*A*), and for proteoglycans alone (*B*). The height of each letter reflects the corresponding amino acid frequency at the indicated positions (P5-P5′) in each group as compared to the human proteome reference set, whereas the color represents its physicochemical properties. The cleaved scissile bond is represented by a dashed line, and amino acid positions upstream and downstream of the cleavage site are numbered as unprimed and primed positions, respectively, according to the Schechter and Berger notation. For the complete list of ECM substrates, see File S4.

Analysis of all known ADAMTS9 cleavages in 48 ECM substrates indicated a modest preference for a tyrosine or arginine at the P1 position, whereas the P1′ pocket has a strong preference for polar amino acids such as serine, threonine or glutamine (Fig. 9*A*). However, when proteoglycans are analyzed separately as substrates, ADAMTS9 has a strong preference for leucine at the P1 position (Fig. 9*B*). In the ADAMTS family, substrate specificities are known to be dictated by exosites located in the ancillary domains (33, 34, 35, 36, 37, 42, 87), which may influence cleavages in GAG-rich regions or unmodified protein regions; glycosylation may also direct proteolytic attack to distinct sites. Nevertheless, our data can be used to design small molecule inhibitors for other pharmaceutical targets, for example ADAMTS4 and ADAMTS5 in osteoarthritis (88), ADAMTS7 in atherosclerosis (89), or ADAMTS8 in pulmonary arterial hypertension (90), which spare ADAMTS9. As ADAMTS4 and ADAMTS5 prefer negatively charged amino acids such as glutamic acid or aspartic acid in P1 and small hydrophobic amino acids in P1′ (38, 52), it should be feasible to design inhibitors of these two proteases sparing ADAMTS9.

In summary, our findings indicate a larger number of ADAMTS cleavage sites in versican isoforms than previously suspected. The combined weight of *in vivo* findings and this study suggests that versican cleavage by ADAMTS9 affects additional sites beside the canonical ones and has significant biological impact. In contrast, ADAMTS9 cleavage of aggrecan and biglycan, which has yet to be shown to have comparable significance, is worthy of further investigation. A practical implication of our work is that the new neo-N- and C- termini at the sites of proteoglycan cleavage by ADAMTS proteases identified in this study may be useful for the generation of neoepitope antibodies (49) and could be measured in tissues and biological fluids for validation as biomarkers of their activity (91).

## Experimental procedures

### Expression and purification of recombinant ADAMTS proteases and proteoglycans

The construct encoding for human ADAMTS9 MDTCS (UniProt ID Q9P2N4, residues 1–877) with an in-frame C-terminal FLAG tag (DYKDDDDK) was custom-synthesized by ThermoFisher Scientific using codons optimized for expression in *Homo sapiens* and subsequently cloned into the pcDNA3.1^(+)^ vector. ADAMTS9 MDTCS EQ containing the E435Q mutation in the active site was generated by site-directed mutagenesis using ADAMTS9 MDTCS as a template. Both constructs were sequenced to confirm that no point mutations were introduced during gene synthesis and PCR.

Full-length ADAMTS1, ADAMTS4, and ADAMTS5 were expressed in human embryonic kidney cells expressing the SV40 large T antigen (HEK293T) and purified by anti-FLAG affinity chromatography as previously reported (35, 37, 58). The same protocol was applied for expression and purification of ADAMTS9 MDTCS E and ADAMTS9 MDTCS EQ. Briefly, ADAMTS constructs were transiently transfected in HEK293T cells using polyethyleneimine (Polysciences Europe GmbH) in the presence of heparin (Sigma-Aldrich, H3393, 200 μg/ml) to release ECM-bound proteins. Seventy-two hours later, the conditioned media was harvested, concentrated on a tangential flow filtration system (Millipore) and incubated with anti-FLAG M2 Affinity resin (Sigma-Aldrich, A2220) before elution with FLAG peptide (Sigma-Aldrich, F3290, 400 μg/ml) and dialysis in TNC-B buffer (20 mM Tris–HCl pH 7.45, 150 mM NaCl, 10 mM CaCl_2_, 0.05% (v/v) Brij-35, 0.02% NaN_3_) to remove the FLAG peptide. Human full-length versican V1 (47) and V2 isoforms (92) were purified by anion exchange chromatography using HiTrap diethylaminoethyl Sepharose (GE Healthcare) (37). Human versican V1-5GAG and full-length biglycan were purified using nickel affinity purification as described before (35, 37).

### QF peptide cleavage assay

QF peptides 1 to 13 were synthesized as before (43), peptide 17 was from Sigma (444282), and all other peptides were custom-synthesized by Bachem (Bubendorf, Germany) (see Table S1 for the peptide sequences). QF peptides were dissolved as 10 mM stocks in dimethylsulfoxide. QF peptide cleavage assays were conducted in 384-well plates (Greiner Bio-One, 784900,) on a SpectraMax i3 Multi-Mode Platform (Molecular Devices), in a final volume of 20 μl at 37 °C. ADAMTS9 MDTCS (10 nM) was incubated with 40 μM of each QF peptide in TNC-B buffer for 24 h at 37 °C. Fluorescent intensities were recorded as appropriate for each fluorophore/quencher pair (*ortho*-amino benzoyl/N-3-[2,4-dinitrophenyl]-L-2,3 diaminopropionyl λ_ex_/λ_em_: 300/430 nm; 7-methoxycoumarin-4-yl/N-3-[2,4-dinitrophenyl]-L-2,3 diaminopropionyl λ_ex_/λ_em_: 328/420 nm; 5,6 fluorescein/N,N,N′,N′-tetramethyl-6-carboxyrhodamine λ_ex_/λ_em_: 485/538 nm), expressed as Relative Fluorescence Units (RFU) and normalized against a blank containing only buffer and substrate. *K*_m_ values were determined at increasing concentrations (0–160 μM) of peptide 14 in the presence of ADAMTS9 MDTCS (0.76 nM) by nonlinear fitting to the Michaelis-Menten equation on GraphPad Prism version 9.2.

v_(0)=V_(max)[S]/(K_(m)+[S])

where *V*_max_ is the maximum initial velocity for the reaction. RFU were converted into product concentration by dividing for the number of RFU generated upon complete digestion of 1.25 μM substrate. *k*_cat_ values were determined by dividing *V*_max_ by the ADAMTS9 concentration. Correction for inner filter effects was performed as before (46). Active site titrations were performed as before (59). ADAMTS9 MDTCS was incubated with TIMP-3 (0–16 nM) for 1 h at 37 °C before addition of QF peptide 14. Relative fluorescence unit values were converted into residual activity by fixing as 100% the activity of the reactions not containing TIMP-3. Following linear fitting of the initial portion of the curve, the active site concentration of ADAMTS9 MDCTS was determined by interpolating the value on the *x* axis, representing the TIMP-3 concentration. The active concentrations of ADAMTS1, ADAMTS4, and ADAMTS5 were determined by titrations with known concentrations of TIMP-3 using QF peptides peptide 15 for ADAMTS1 and ADAMTS4, and peptide one for ADAMTS5 (35, 37).

### ADAMTS9 autolysis digests

ADAMTS9 MDTCS E and EQ (12.5 nM) were separately incubated at 37 °C for 2 h before addition of ethylenediaminetetraacetic acid (EDTA, 25 mM) to terminate the digest.

### Proteoglycan cleavage assays

In house-purified recombinant human versican V1 (100 nM), V1-5GAG (100 nM), V2 (1000 nM) and biglycan (2000 nM), and aggrecan from bovine articular cartilage (667 nM, Sigma-Aldrich, A1960), were digested with various concentrations of ADAMTS9 MDTCS, ADAMTS1, ADAMTS4, and ADAMTS5, in TNC-B buffer at 37 °C as stated in the Results sections. For inhibition experiments, ADAMTS9 MDTCS (25 nM) was preincubated with recombinant TIMP-1, TIMP-2, TIMP-3 or TIMP-4 (each at 500 nM, Bio-Techne Ltd, 970-TM, 971-TM, 973-TM, and 974-TSF) in TNC-B for 1h at 37°C before addition of V1-5GAG (100 nM). At different time points, aliquots were removed and reactions were stopped with EDTA (25 mM) in deglycosylation buffer (50 mM sodium acetate, 25 mM Tris HCl pH 8.0) containing 0.1 U/ml chondroitinase ABC (AMS Biotechnology Europe, AMS.E1028–02) for 16 h at 37 °C. In aggrecan digests, 0.1 U/ml endo-beta galactosidase (Sigma, G6920) was added to remove keratan sulfate chains. Samples were heat-denatured, electrophoresed under reducing conditions (5% β-mercaptoethanol) in 4 to 12% Bolt Bis-Tris Plus Mini Protein Gels (Fisher Scientific), and transferred to nitrocellulose membranes using Trans-Blot Turbo Transfer System (Bio-Rad Laboratories). Non-specific sites were blocked with 3% (w/v) bovine serum albumin in phosphate buffered saline (PBS) for 1 h at room temperature. Membranes were incubated overnight at 4 °C with the following antibodies, all prepared in 0.5% bovine serum albumin/PBS: rabbit polyclonal anti-Vc, recognizing the versican sequence ^432^TVPKDPEAAEARRG^445^ spanning the E441-A442 cleavage site in V1 GAGβ domain (1 μg/ml) (47); rabbit polyclonal anti-DPEAAE neoepitope (Life Technologies, PA1-1748A, 2 μg/ml) which only detects versikine, the N-terminal versican fragment generated after proteolysis at E441-A442 in GAGβ; mouse monoclonal anti-ARGSV neoepitope (BC3) recognizing aggrecanase cleavage at E392-A393 (Life Technologies, MA3-16888, 4 μg/ml); mouse monoclonal 2B6, recognizing the CS stubs remaining on proteoglycans after chondroitinase ABC treatment of proteoglycans with chondroitinase ABC (AMS Biotechnology Europe, 270432-CS, 1:100); goat polyclonal anti-biglycan (Bio-Techne Ltd, AF2667, 0.4 μg/ml). The membranes were then washed three times with PBS containing 0.1% TWEEN 20 (Sigma), (PBS-T) for 10 min each, before being placed in appropriate horseradish peroxidase (HRP)-conjugated secondary antibody (Agilent Technologies) for 1 h at room temperature. Following three additional washing with PBS-T, membranes were covered in enhanced HRP chemiluminescent substrate (Millipore-Merck, WBKLS0050) for 4 min before being scanned on a Licor Odyssey imager. Band intensity was quantified using the ImageJ software tool. Signals were normalized to the total protein loaded on each lane. All quantifications were performed on images taken with the same exposure settings and without post-image processing. Wherever indicated, SDS-PAGE gels were stained with Coomassie Brilliant Blue stain (Imperial Stain, ThermoFisher Scientific, 24615) for at least 2 h, before being extensively destained with distilled water.

Figures for unprocessed blots and gels are also included in Figs. S6–S16 with highlighted regions (in red boxes) corresponding to the data reported in the main manuscript figures.

### Versikine ELISA

ADAMTS9 MDTCS (25 nM) was incubated with 50 nM versican V1 or V1-5GAG at 37 °C in TNC-B buffer. At different time points (0–120 min), an aliquot of each sample was removed, and reaction was stopped with the addition of EDTA buffer. The amount of versikine neoepitope generated in each reaction was measured by sandwich ELISA as previously reported (37, 50). Briefly, 96-well Maxisorp plates (Nunc) were coated with 5 μg/ml anti-DPEAAE neoepitope antibody in 3% BSA/PBS (16 h, 4 °C), and blocked with 3% BSA/PBS (2 h, at room temperature, RT). Samples from digestion reactions were diluted in 3% BSA/PBS and incubated for 2 h at RT. Bound DPEAAE-containing versican fragments were detected using mouse anti-versican monoclonal antibody (Abcam, ab171887, 3 μg/ml in 0.5% BSA/PBS, 1.5 h at RT) followed by HRP-conjugated anti-mouse antibodies (2.4 μg/ml in 0.5% BSA/PBS, 1 h at RT). Each step was followed by three washes with PBS-T. Finally, signals were developed by addition of o-phenylenediamine dihydrochloride for 10 min and reactions were stopped with 2 M H_2_SO_4_. The absorbance was read at 492 nm.

### Label-free quantitative proteomics analysis by LC-MS/MS and z-score analysis

Proteoglycan cleavage assays were carried out as described above using E and EQ constructs of ADAMTS1 (140 nM), ADAMTS4 (5 nM), ADAMTS5 (5 nM), and ADAMTS9 MDTCS (12.5 nM) for 2 h at 37 °C. Under these conditions and as assessed by immunoblots and CBB, proteolysis was not complete, which allowed identification of the most efficient proteolytic events. Four micrograms of ADAMTS-digested proteoglycans were vacuum centrifuged to near dryness and resuspended in 6 M urea, 25 mM ammonium bicarbonate. Proteins were reduced at 37 °C for 30 min with 10 mM dithiothreitol followed by alkylation at room temperature in the dark for 20 min with 32 mM iodoacetamide. Excess iodoacetamide was quenched with an additional 25 mM dithiothreitol. The urea was then diluted 10-fold in 25 mM ammonium bicarbonate. Proteins were digested overnight with trypsin (Promega, V5280) or GluC (Promega, V1651) at 37 °C at a 1:50 or 1:25 protease to substrate ratio, respectively.

Peptides were desalted using Sep-Pak Vac 1CC desalt columns (Waters, WAT054955) activated with 100% acetonitrile, equilibrated and desalted with 0.1% trifluoracetic acid, and eluted in 30:70 0.1% trifluoracetic acid: 100% acetonitrile. Eluted peptides were vacuum centrifuged until dry and resuspended in 0.1% formic acid for LC-MS/MS analysis. LC-MS/MS analysis was performed on a Bruker TimsTof Pro2 Q-Tof mass spectrometry system operating in positive ion mode, coupled with a CaptiveSpray ion source (both from Bruker Daltonik GmbH, Bremen). The HPLC column was a Bruker 15 cm × 75 μm id C18 ReproSil AQ, 1.9 μm, 120 Å reversed-phase capillary chromatography column. One microliter volumes of the extract were injected and the peptides eluted from the column by an acetonitrile/0.1% formic acid gradient at a flow rate of 0.3 μl/min were introduced into the source of the mass spectrometer on-line. The digests were analyzed using a Parallel Accumulation–Serial Fragmentation DDA method to select precursor ions for fragmentation with a TIMS-MS scan followed by 10 PASEF MS/MS scans. The TIMS-MS survey scan was acquired between 0.60 and 1.6 Vs/cm^2^ and 100–1700 m/z with a ramp time of 166 ms. The total cycle time for the PASEF scan was 1.2 s and the MS/MS experiments were performed with a collision energy between 20 eV (0.6 Vs.cm^2^) to 59 eV (1.6 Vs/cm^2^). Precursors with 2 to 5 charges were selected with the target value set to 20,000 a.u and intensity threshold to 2500 a.u. Precursors were dynamically excluded for 0.4 s.

LC-MS/MS raw data were searched against both the full human database with isoforms (August 2023) supplemented with the exact protein sequences of the recombinant proteases and substrates (ADAMTS1/ADAMTS4/ADAMTS5/ADAMTS9, biglycan, versican V1, and versican V2) and a limited database of just the protein sequences of the recombinant proteases and substrates using the Fragpipe (V22.0) interface for MsFragger (V4.1) database search engine software (University of Michigan, https://doi.org/10.1038/nmeth.4256). The database search was performed using label-free ion-mobility settings with a semi-tryptic/GluC digestion, allowing for up to two missed cleavages, peptide length between 6 to 75 amino acids, at least one unique peptide identified per protein (the number of razor peptides was not specified), and including carbamidomethylation of cysteine as a fixed modification and methionine or proline oxidation and N-terminal glutamine to pyro-glutamate cyclization, or acetylation as variable modifications. Full scan mass tolerance was set to 10 ppm and fragment mass tolerance to 0.02 Da. Match between runs was enabled, peptide spectral matches were validated using percolator set to a false discovery rate of 1%, and LFQ was performed at the peptide level using IonQuant (V 1.10.27). Peptide intensities were compared between each protease (E/EQ) pair with statistical tests including *z*-score analysis and abundance outlier analysis using Microsoft 365 Excel. *Z*-scores were calculated as previously described (38) and abundance outliers were calculated using the 1.5 interquartile rule based on the abundance of all peptides quantified in each respective sample pair. Graphs were created using GraphPad Prism (version 10).

The mass spectrometry proteomics data have been deposited to the ProteomeXchange Consortium *via* the PRIDE (93) partner repository with the dataset identifier PXD061892.

All proteins and peptides identified are provided in File S1 (proteins), File S2 and File S3 (peptides).

### Determination of ADAMTS9 cleavage site specificity

A list of ADAMTS9 substrates was initially downloaded from the MEROPS database (https://www.ebi.ac.uk/merops/index.shtml) (94) under identifier M12.021 and manually analyzed to remove non-ECM substrates and redundant cleavage sites in orthologous proteins. Cleavage sites were manually annotated to reflect UniProt identifiers instead of traditional nomenclature and original references were inspected. Where appropriate, cleavage sites not attributed to ADAMTS9 in the original papers were removed and missing cleavage sites were added. Finally, the cleavage sites identified in the present study were added. The final list (File S4), comprising 210 distinct cleavage sites in 48 ECM substrates, was uploaded on the IceLogo web server (https://iomics.ugent.be/icelogoserver) (95) for generation of logos against a precompiled Swiss-Prot composition reference set from *H. sapiens*. The following parameters were selected: scoring system: percentage (*i.e.*, comparing the frequency percentage of an amino acid at a certain location in the multiple sequence alignment and the reference set); *p* value: 0.05.

### Statistical analysis

Statistical analysis was performed on GraphPad Prism software (version 8.4) using one-way ANOVA with Dunn’s multiple comparisons. *p* < 0.05 was considered statistically significant.

## Data availability statement

All data described in this study are contained within the manuscript.

## Supporting information

This article contains Figs. S1–S16, Table S1, and Files S1–S4.

## Conflict of interest

The authors declare that they have no conflicts of interest with the contents of this article.
