# Supplementary material for: Characterization of ADAMTS9 proteoglycanase activity: Comparison with ADAMTS1, ADAMTS4, and ADAMTS5
Source: J Biol Chem. 2025 May 29;301(7):110301. doi: 10.1016/j.jbc.2025.110301 (PMC12226136; doi:10.1016/j.jbc.2025.110301)
Supplement: Supporting Data [file mmc5.docx]

**SUPPORTING INFORMATION**

**Characterization of ADAMTS9 proteoglycanase activity: comparison with ADAMTS1, ADAMTS4 and ADAMTS5**

Daniel R. Martin,^1≠^ Gemma Sardelli,^2,3≠^ Tina Burkhard,^3^ Milan M. Fowkes,^4^ Alexander F. Minns,^3^ Roberta Moschini,^2^ Antonella Del Corso,^2^ Rens de Groot,^5^ Suneel S. Apte,^1^* and Salvatore Santamaria^3,6^*

^1^Department of Biomedical Engineering, Cleveland Clinic Research, Cleveland, OH 44195, USA;

^2^Department of Biology, Biochemistry Unit, University of Pisa, 56123 Pisa, Italy

^3^Department of Biochemical Sciences, School of Biosciences, Faculty of Health and Medical Sciences, Edward Jenner Building, University of Surrey, Guildford, Surrey GU2 7XH, United Kingdom;

^4^Centre for Medicines Discovery, Nuffield Department of Medicine Research Building, University of Oxford, Oxford, OX3 7FZ, United Kingdom;

^5^Institute of Cardiovascular Science, University College London, 51 Chenies Mews, London WC1E 6HX, United Kingdom;

^6^Department of Immunology and Inflammation, Imperial College London, Du Cane Road, London W12 0NN, United Kingdom

^≠^these authors contributed equally to the work

*to whom the correspondence should be addressed: Suneel S. Apte, Department of Biomedical Engineering, Cleveland Clinic Research, Cleveland, OH 44195, USA, [aptes@ccf.org](mailto:aptes@ccf.org);

Salvatore Santamaria, Department of Biochemical Sciences, School of Biosciences, Faculty of Health and Medical Sciences, Edward Jenner Building, University of Surrey, Guildford, Surrey GU2 7XH, United Kingdom, [s.santamaria@surrey.ac.uk](mailto:s.santamaria@surrey.ac.uk); Department of Immunology and Inflammation, Imperial College London, Du Cane Road, London W12 0NN, United Kingdom, [s.santamaria@imperial.ac.uk](mailto:s.santamaria@imperial.ac.uk)

**This Supporting Information contains Supporting Figures 1-16 and Supporting Table 1.**

**Supporting Figure 1:** Plots of log2 peptide abundance *versus* *z*-score for fully tryptic (**A**) and fully GluC (**B**) ADAMTS9 MDTCS auto-digests. Dashed blue and red lines indicate the thresholds for *z*-score and log2 peptide abundance, respectively.

**
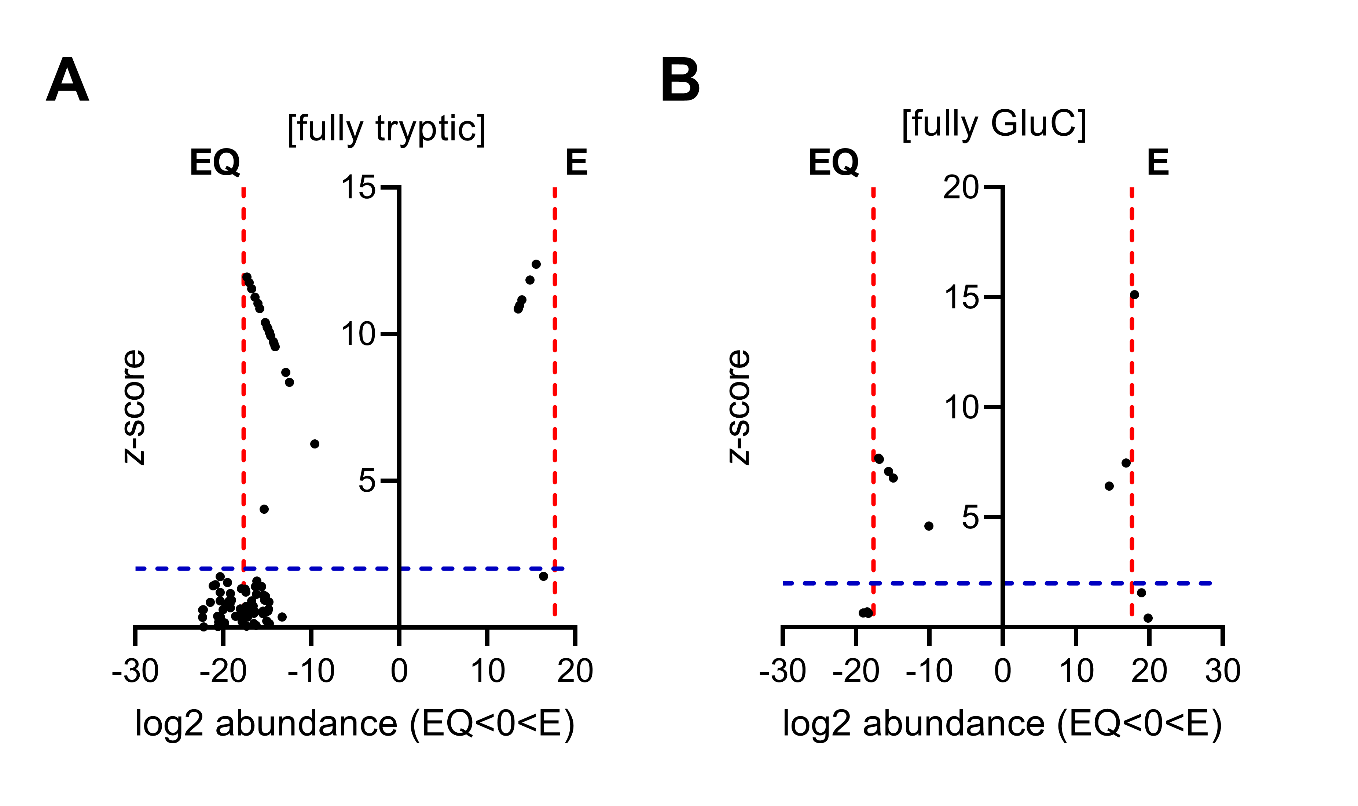
**

**Supporting Figure 2:** Donut charts showing the lower prevalence of predicted tryptic (R, K) and GluC (E, D) cleavage sites in human versican V1 (UniProt ID: P13611-2), versican V2 (UniProt ID: P13611-3), and aggrecan (UniProt ID: P16112), compared to biglycan (UniProt ID: P21810) and the rest of the human proteome.


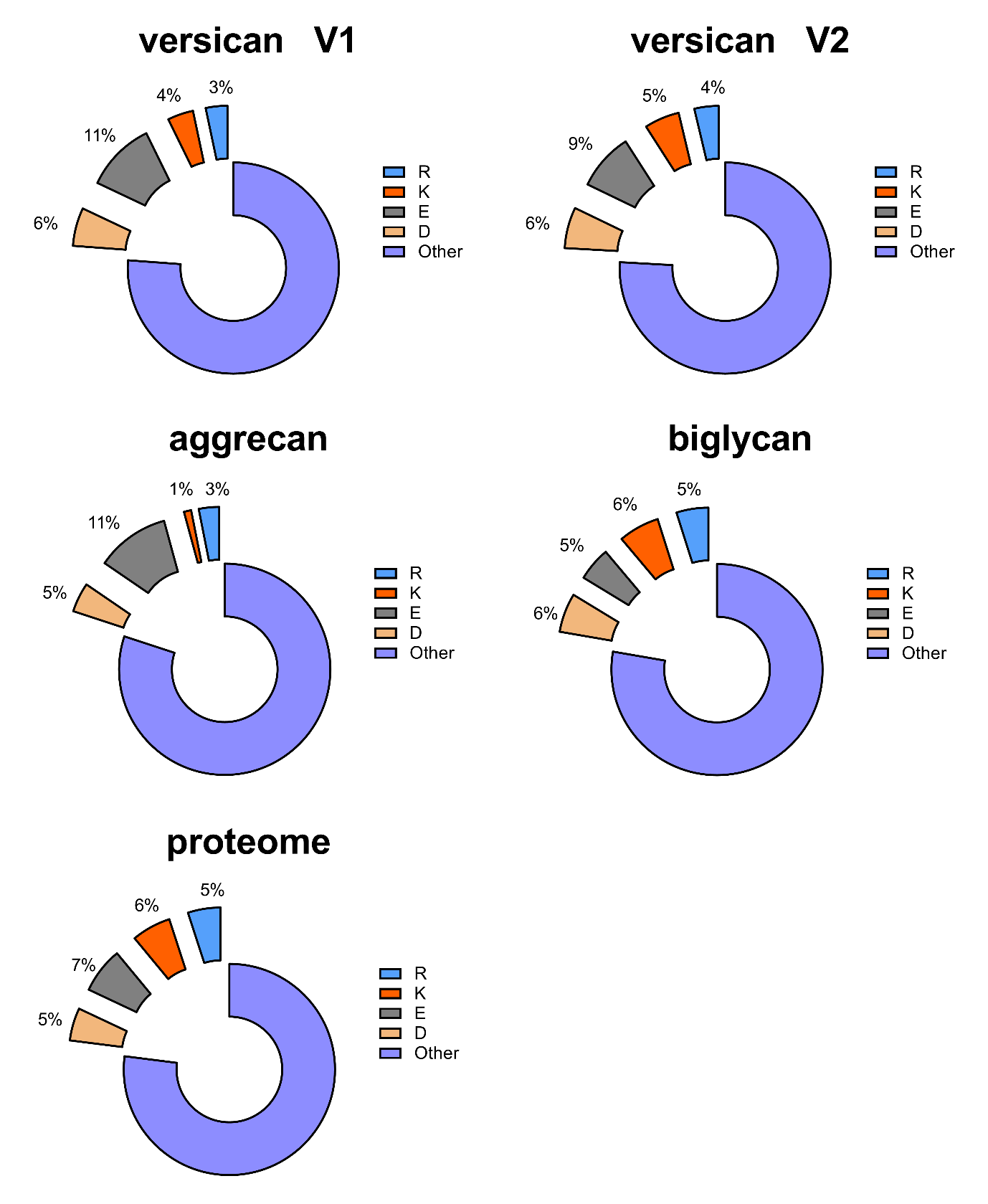


**Supporting Figure 3.** Density of predicted cleavages for GluC (red lines) and trypsin (black lines) in human versican V1, versican V2, aggrecan (hAggrecan), biglycan, and bovine aggrecan (bAggrecan). Note the relative paucity of predicted tryptic sites in the GAG domains of both versican isoforms and aggrecan. CS, chondroitin-sulfate rich domain; G1 and G3, globular domains; IGD, interglobular domain; KS, keratan-sulfate rich domain; LRR, leucine-rich repeats.

**
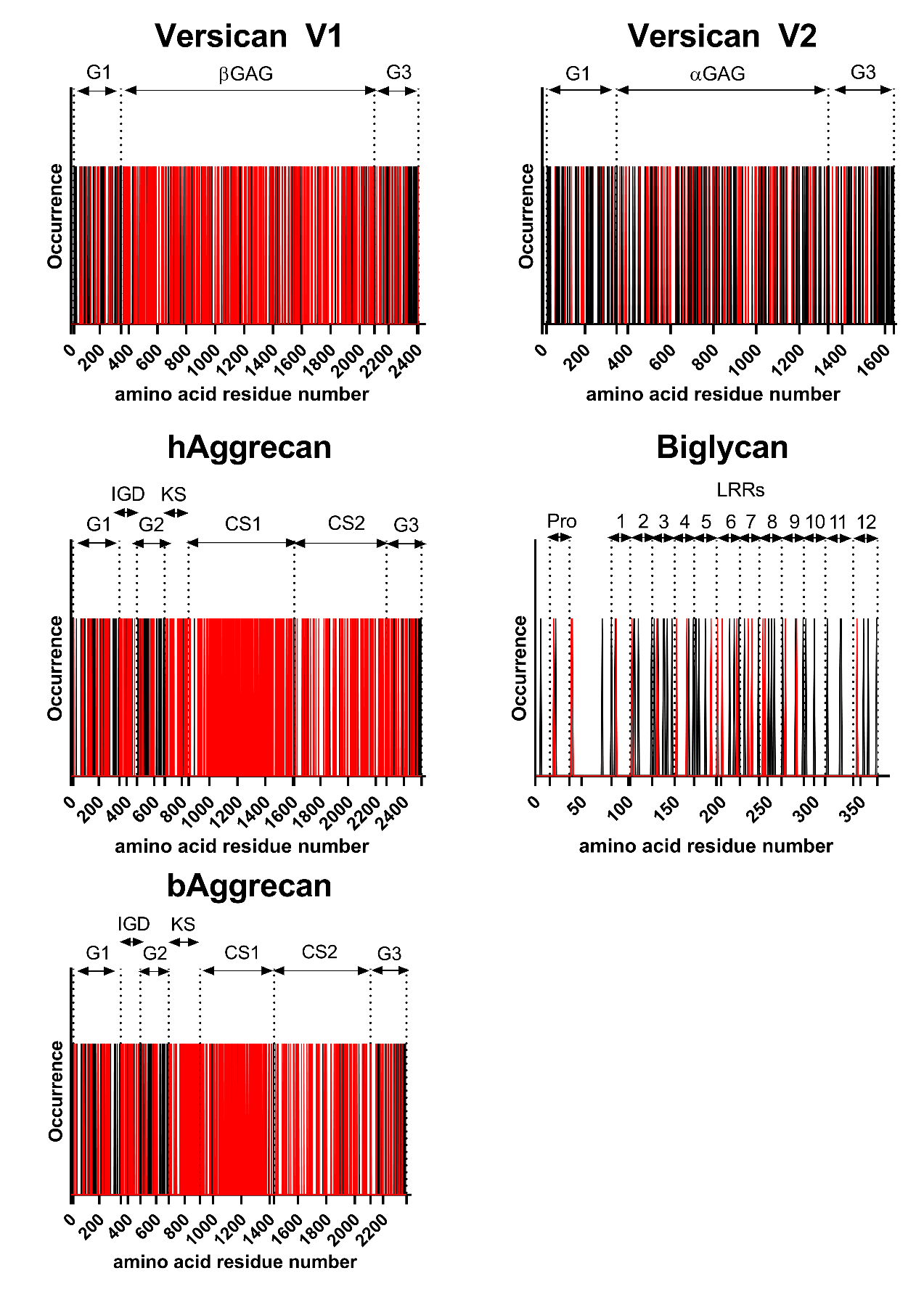
**

**Supporting Figure 4.** Plots of log2 peptide abundance *versus* *z*-score for fully GluC (A,C,E) and fully tryptic (B, D, F) versican V2 peptides generated by ADAMTS1 (A-B), ADAMTS4 (C-D), and ADAMTS5 (E-F). Dashed blue and red lines indicate the thresholds for *z*-score and log2 peptide abundance, respectively. Numbers indicate the first and last amino acid of peptides significantly more abundant in the EQ digests.

**
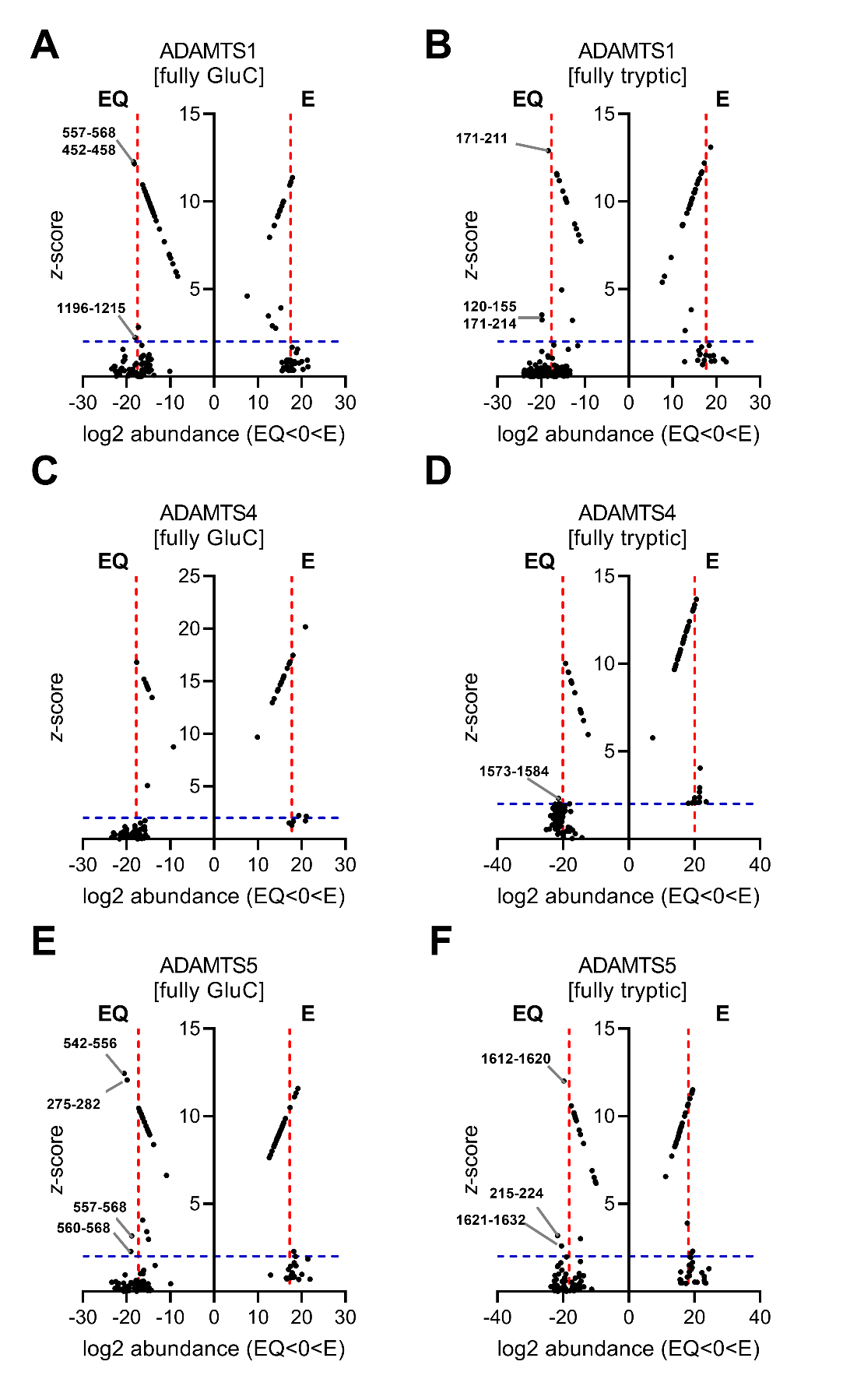
**

**Supporting Figure 5.** Schematic summarizing bovine aggrecan LC-MS/MS coverage without **(A)** and with **(B)** deglycosylation by chondroitinase ABC. Prior to deglycosylation, coverage was 29% and improved marginally to 31% following chondroitinase ABC and keratanase treatment. The identified and unidentified sequences are shown in red, and black, respectively. AA#, residue number.

**
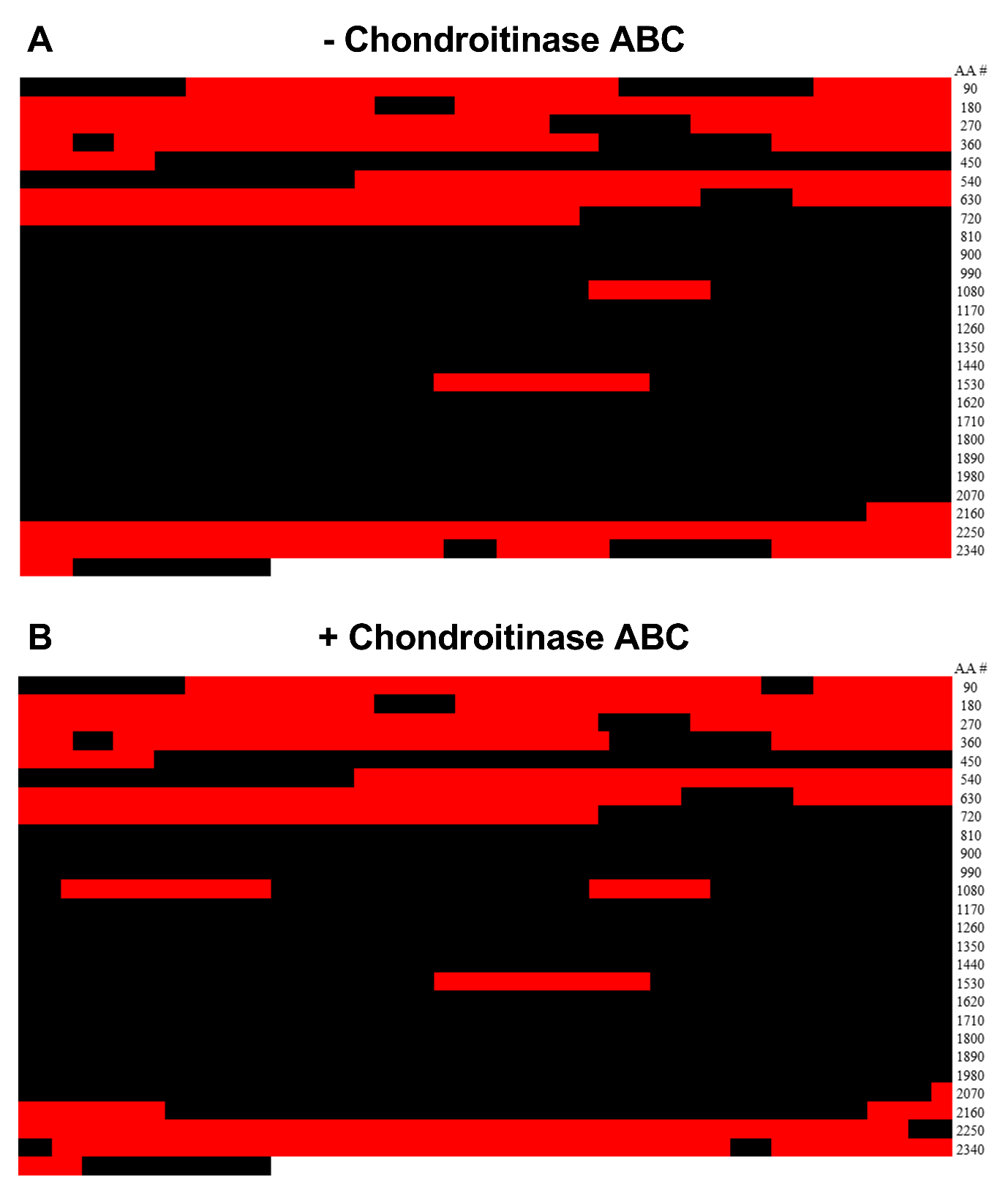
**

**Supporting Figure 6:** Full Coomassie Brilliant Blue staining corresponding to the gel reported in Figure 1B. Highlighted region (in red box) corresponds to the data reported in the main manuscript figure.


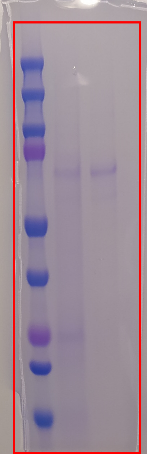


**Supporting Figure 7:** Full anti-Vc immunoblot corresponding to Figure 4C. Highlighted region (in red box) corresponds to the data reported in the main manuscript figure.

**
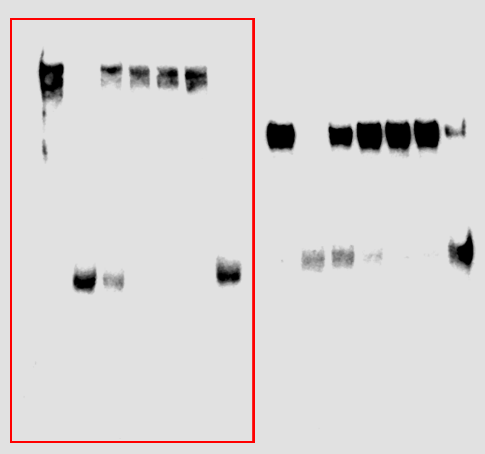
**

**Supporting Figure 8:** Full anti-DPEAAE immunoblot corresponding to Figure 4, panels C and D. Highlighted region (in red box) corresponds to the data reported in the main manuscript figure.


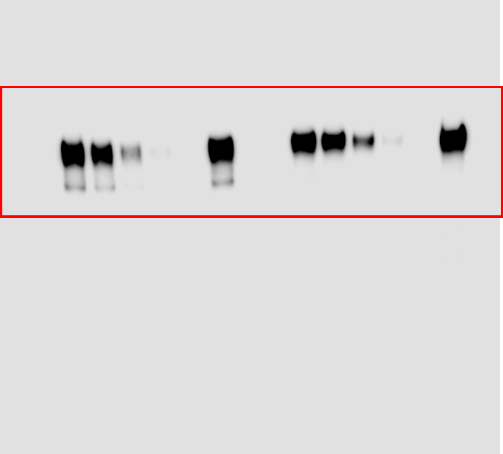


**Supporting Figure 9:** Full anti-Vc immunoblot corresponding to Figure 4D. Highlighted region (in red box) corresponds to the data reported in the main manuscript figure.


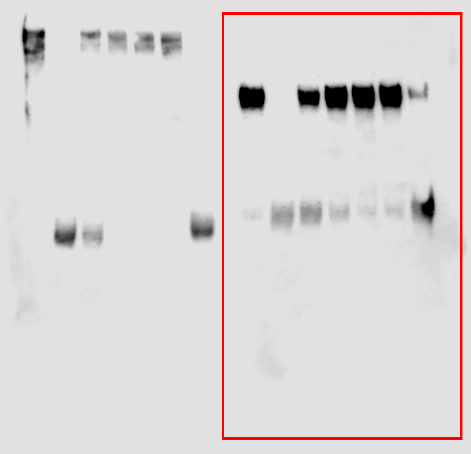


**Supporting Figure 10:** Full anti-DPEAAE immunoblot corresponding to Figure 4E. Highlighted region (in red box) corresponds to the data reported in the main manuscript figure.


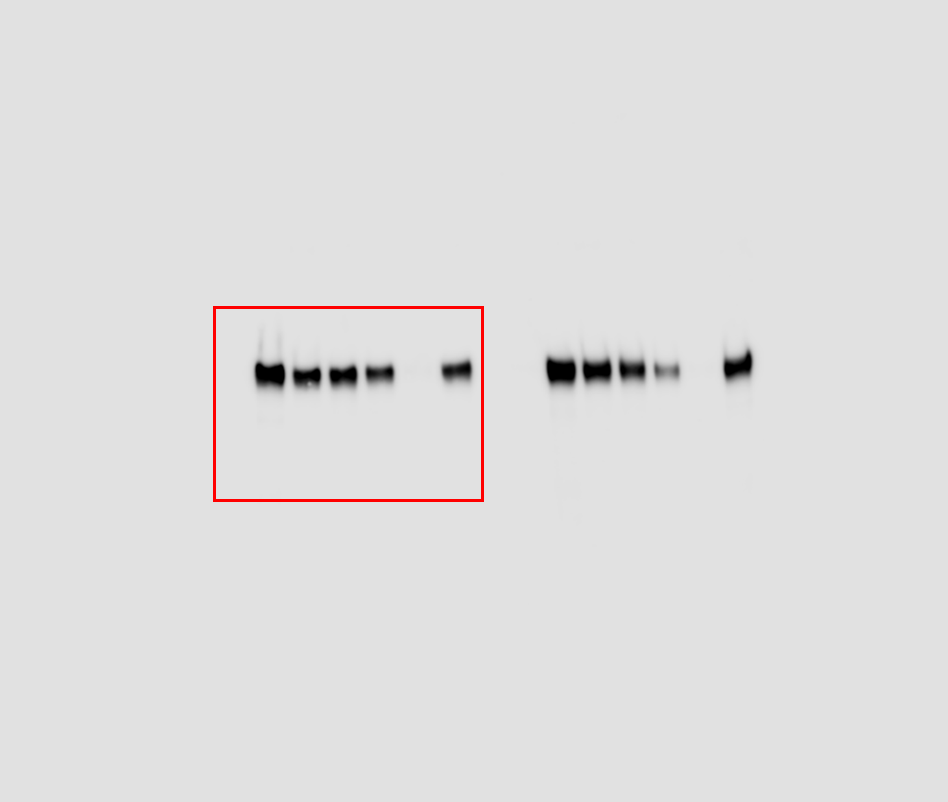


**Supporting Figure 11:** Full Coomassie Brilliant Blue staining for the gel reported in Figure 6A. Highlighted region (in red box) corresponds to the data reported in the main manuscript figure.

**
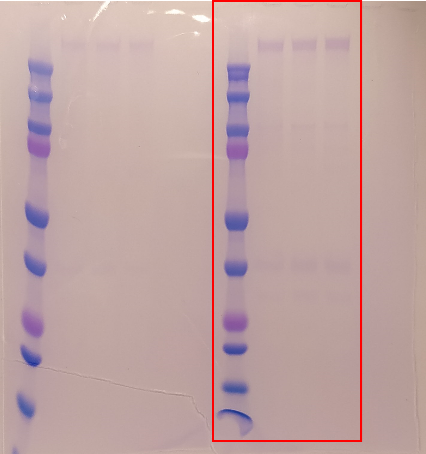
**

**Supporting Figure 12:** Full Coomassie Brilliant Blue staining for the gel reported in Figure 6B. Highlighted region (in red box) corresponds to the data reported in the main manuscript figure.


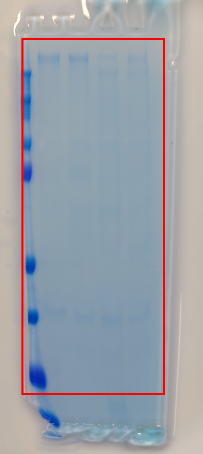


**Supporting Figure 13:** Full anti-ARGSV immunoblot corresponding to Figure 7A. Highlighted region (in red box) corresponds to the data reported in the main manuscript figure.


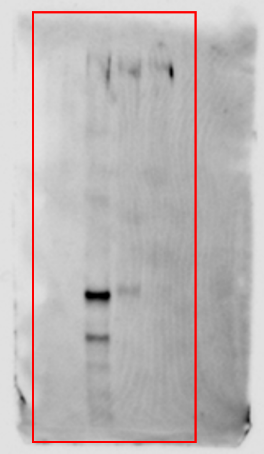


**Supporting Figure 14:** Full anti-CS immunoblot corresponding to Figure 7B. Highlighted region (in red box) corresponds to the data reported in the main manuscript figure.

**
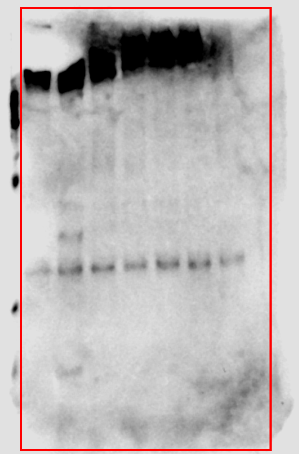
**

**Supporting Figure 15:** Full Coomassie Brilliant Blue staining for the gel reported in Figure 8A. Highlighted region (in red box) corresponds to the data reported in the main manuscript figure.

**
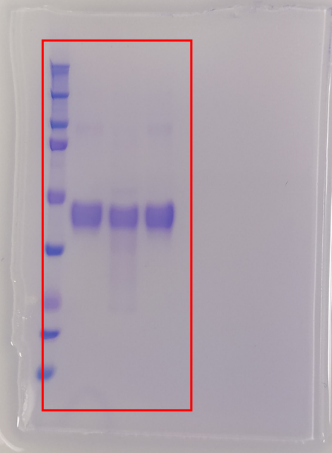
**

**Supporting Figure 16:** Full anti-biglycan immunoblot corresponding to Figure 8B. Highlighted region (in red box) corresponds to the data reported in the main manuscript figure.


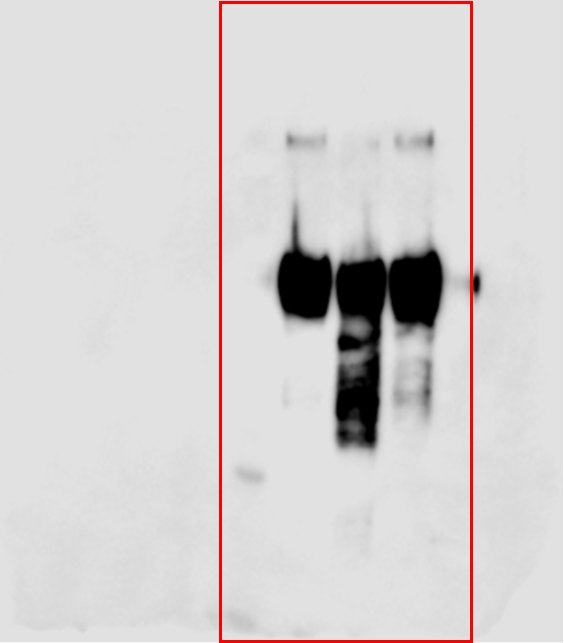


**Supporting Table 1: Quenched-fluorescent peptides tested as ADAMTS9 substrates.** Abz, *ortho*-amino benzoyl; Dpa, N-3-[2,4-dinitrophenyl]-L-2,3 diaminopropionyl; FAM, 5,6 fluorescein; Mca, 7-methoxycoumarin-4-yl; MMP, matrix metalloproteinase; TAMRA, N,N,N’,N’-tetramethyl-6-carboxyrhodamine; Y(NO_2_), 3-nitro-L-tyrosine. Non-canonical amino acids are indicated as follows: O = ornithine, B = 2,4-diaminobutyric acid, J = β-cyclopropyl-alanine, Z = 4-thiazolyl-alanine, U = 2,3-diaminopropionic acid, *AEEA* = 8-amino-3,6-dioxaoctanoic acid.

| **Peptide** | **Sequence** | **Cleaving protease** | **Reference** |
| --- | --- | --- | --- |
| 1 | Abz-TESESRGAIY-Dpa-KK-NH_2_ | ADAMTS5 | [40] |
| 2 | KY(NO_2_)TESESRGK(Abz)IYYKKG | ADAMTS5 | [43] |
| 3 | KY(NO_2_)SEGESRGK(Abz)JYFKKG | ADAMTS5 | [43] |
| 4 | KY(NO_2_)TEGESRGK(Abz)JZYKKG | ADAMTS5 | [43] |
| 5 | KY(NO_2_)SENESRGK(Abz)IYYKKG | ADAMTS5 | [43] |
| 6 | WYRGRL-*AEEA*-KY(NO_2_)TESESRGK(Abz)IYYKKG | ADAMTS5 | [43] |
| 7 | WYRGRL-*AEEA*-KY(NO_2_)NDTESOAK(Abz)AHFUKG | ADAMTS5 | [43] |
| 8 | KY(NO_2_)TENESRGK(Abz)IYYKKG | ADAMTS5 | [43] |
| 9 | KY(NO_2_)QDSESBAK(Abz)JHYUKG | ADAMTS5 | [43] |
| 10 | KY(NO_2_)TETESOAK(Abz)IYFKKG | ADAMTS5 | [43] |
| 11 | KY(NO_2_)SESESRGK(Abz)IYYKKG | ADAMTS5 | [43] |
| 12 | KY(NO_2_)NDTESOAK(Abz)AHFUKG | ADAMTS5 | [43] |
| 13 | KY(NO_2_)NESESKAK(Abz)VHYKKG | ADAMTS5 | [43] |
| 14 | FAM-AELQGRPISIAK-TAMRA | ADAMTS4 | [44] |
| 15 | FAM-AELNGRPISIAK-TAMRA | ADAMTS1/4 | [35] |
| 16 | FAM-EAAARAEAAAK-TAMRA | ADAMTS4/5/7 | [39] |
| 17 | Mca-KPLGL(Dpa)-AR | MMPs/ADAMs | [46] |
